# SlZHD17 is involved in the control of chlorophyll and carotenoid metabolism in tomato fruit

**DOI:** 10.1038/s41438-021-00696-8

**Published:** 2021-12-01

**Authors:** Yuan Shi, Xiaoqin Pang, Wenjing Liu, Rui Wang, Deding Su, Yushuo Gao, Mengbo Wu, Wei Deng, Yudong Liu, Zhengguo Li

**Affiliations:** 1 Key Laboratory of Plant Hormones and Development Regulation of Chongqing, School of Life Sciences, Chongqing University, 401331 Chongqing, China; 2 Center of Plant Functional Genomics, Institute of Advanced Interdisciplinary Studies, Chongqing University, 401331 Chongqing, China

**Keywords:** Plant molecular biology, Transcriptional regulatory elements

## Abstract

Chlorophylls and carotenoids are essential and beneficial substances for both plant and human health. Identifying the regulatory network of these pigments is necessary for improving fruit quality. In a previous study, we identified an R2R3-MYB transcription factor, SlMYB72, that plays an important role in chlorophyll and carotenoid metabolism in tomato fruit. Here, we demonstrated that the SlMYB72-interacting protein SlZHD17, which belongs to the zinc-finger homeodomain transcription factor family, also functions in chlorophyll and carotenoid metabolism. Silencing *SlZHD17* in tomato improved multiple beneficial agronomic traits, including dwarfism, accelerated flowering, and earlier fruit harvest. More importantly, downregulating *SlZHD17* in fruits resulted in larger chloroplasts and a higher chlorophyll content. Dual-luciferase, yeast one-hybrid and electrophoretic mobility shift assays clarified that SlZHD17 regulates the chlorophyll biosynthesis gene *SlPOR-B* and chloroplast developmental regulator *SlTKN2* in a direct manner. Chlorophyll degradation and plastid transformation were also retarded after suppression of *SlZHD17* in fruits, which was caused by the inhibition of *SlSGR1*, a crucial factor in chlorophyll degradation. On the other hand, the expression of the carotenoid biosynthesis genes *SlPSY1* and *SlZISO* was also suppressed and directly regulated by SlZHD17, which induced uneven pigmentation and decreased the lycopene content in fruits with *SlZHD17* suppression at the ripe stage. Furthermore, the protein–protein interactions between SlZHD17 and other pigment regulators, including SlARF4, SlBEL11, and SlTAGL1, were also presented. This study provides new insight into the complex pigment regulatory network and provides new options for breeding strategies aiming to improve fruit quality.

## Introduction

Fleshy fruits are major sources of necessary nutrients in many diets worldwide. One of the most dramatic events during fleshy fruit ripening is the change in color. This process occurs with the degradation of chlorophyll accompanied by the accumulation of carotenoids, as well as other beneficial substances, including anthocyanins and flavonoids^1^. Key structural genes in chlorophyll and carotenoid biosynthesis have been identified, and altering the expression of structural genes significantly affects pigment accumulation in plants^2,3^. Uncovering the regulators of pigment metabolism is crucial for breeding good-quality fruits.

Recently, several regulatory components that control these biological processes have been identified. The dominant *uniform ripening* (*U*) locus encodes a GOLDEN2-LIKE (SlGLK2) transcription factor and is crucial for the distribution and intensity of plastids and chlorophylls in tomato fruit^4,5^. The *ARABIDOPSIS PSEUDO RESPONSE REGULATOR2-LIKE* gene (*SlAPRR2-like*) is a *SlGLK2*-related gene. The number and area of plastids in fruit overexpressing this gene were increased, and the levels of chlorophyll in unripe fruit and carotenoids in ripe fruit were both improved^6^. The high-pigment mutants *highpigment1* (*hp1*) and *hp2*, which harbor lesions in the *UV-DAMAGED DNA BINDING PROTEIN1* (*DDB1*) and *DEETIOLATED1* (*DET1*) genes, respectively, showed increased plastid number and size, and the levels of chlorophyll and carotenoids were both enhanced in the fruits^7–9^. A CULLIN4 (CUL4) protein assembled with DDB1 and DET1 to form a ubiquitin ligase complex that degraded GLK2^10^. The class I KNOTTED1-like homeobox transcription factors SlTKN2 and SlTKN4 act upstream of *SlGLK2* and/or *SlAPRR2-like* to establish a gradient of chloroplast development^11^. In addition, downregulation of the transcription factor *AUXIN RESPONSE FACTOR 4* (*SlARF4*, also named *DR12*) in tomato increased the number of chloroplasts at the immature stage in dark-green fruit and exhibited blotchy ripening; this effect seems to be caused by the upregulation of *SlGLKs*^12,13^. Both SlARF6A and SlARF10 could directly bind to the *SlGLK1* promoter and positively regulate chloroplast development and chlorophyll accumulation^14,15^. Moreover, silencing *BEL1-LIKE HOMEODOMAIN 11* (*SlBEL11*) increased the numbers of thylakoids and chloroplasts in green fruit^16^. Downregulation of *BEL1-LIKE HOMEODOMAIN 4* (*SlBL4*) resulted in a slightly darker green fruit, and increased thylakoids and chloroplasts were also found in *SlBL4* RNAi fruit^17^. In addition, tomato plants with reduced *SlTAGL1* mRNA produced yellow–orange fruit with reduced carotenoids and suppressed chlorophyll breakdown^18,19^. These studies showed that the processes of pigment metabolism and plastid transformation were modulated by multiple types of regulators; however, these studies mainly focused on a few transcription factor families. Identifying additional transcription factors that regulate these processes will broaden our knowledge in this field.

Zinc-finger homeodomain (ZHD) proteins belong to the HD superfamily, which contains a C2H2-type zinc-finger motif (ZF) and a conserved DNA-binding homeodomain (HD). ZHD proteins can bind to DNA sequences with a core consensus of NNATTA^20^. In recent years, the functions of some ZHD members from different species have been reported. A *Flaveria trinervia* ZHD was identified as a potential regulator of the *C4 phosphoenolpyruvate carboxylase* (*PEPCase*) gene^21^. Soybean *GmZF-HD1* and *GmZF-HD2* were induced by pathogen inoculation, and they could bind to the promoter region of a *calmodulin isoform 4* (*GmCaM4*) gene^22^. Arabidopsis ZFHD1 can bind to the *EARLY RESPONSE TO DEHYDRATION STRESS 1* (*ERD1*) gene promoter, and ZFHD1 can interact with NAC proteins to improve drought stress tolerance^23^. ARABIDOPSIS THALIANA HOMEOBOX 25 (ATHB25/ZFHD2/ZHD1) positively regulated the *GIBBERELLIC ACID3-OXIDASE2* (*GA3ox2*) gene, thus influencing GA biosynthesis, which determined seed longevity^24^. To date, except for these studies, no other ZHDs have been reported. In tomato, 22 *SlZHD* genes have been identified^25^, but no functional study has been reported in this species.

In our previous study, downregulation of *SlMYB72* resulted in uneven fruit coloration, increased chlorophyll accumulation and chloroplast development in immature fruit, and decreased lycopene content in ripe red fruit^26^. Here, we identified a SlMYB72-interacting protein, SlZHD17. Silencing *SlZHD17* resulted in disordered chlorophyll and carotenoid biosynthesis and retarded plastid transformation in tomato fruit. SlZHD17 directly regulated the genes involved in chlorophyll metabolism, chloroplast development and carotenoid biosynthesis. In addition, protein–protein interactions between SlZHD17 and the SlARF4, SlBEL11, and SlTAGL1 proteins were also identified. Our results provide new insight into the involvement of SlZHD17 in the regulation of pigment metabolism and reveal that the tomato ZHD transcription factor plays a crucial role in fruit development and ripening.

## Results

### SlMYB72 interacts with the zinc-finger homeodomain transcription factor SlZHD17

**Fig. 1:**
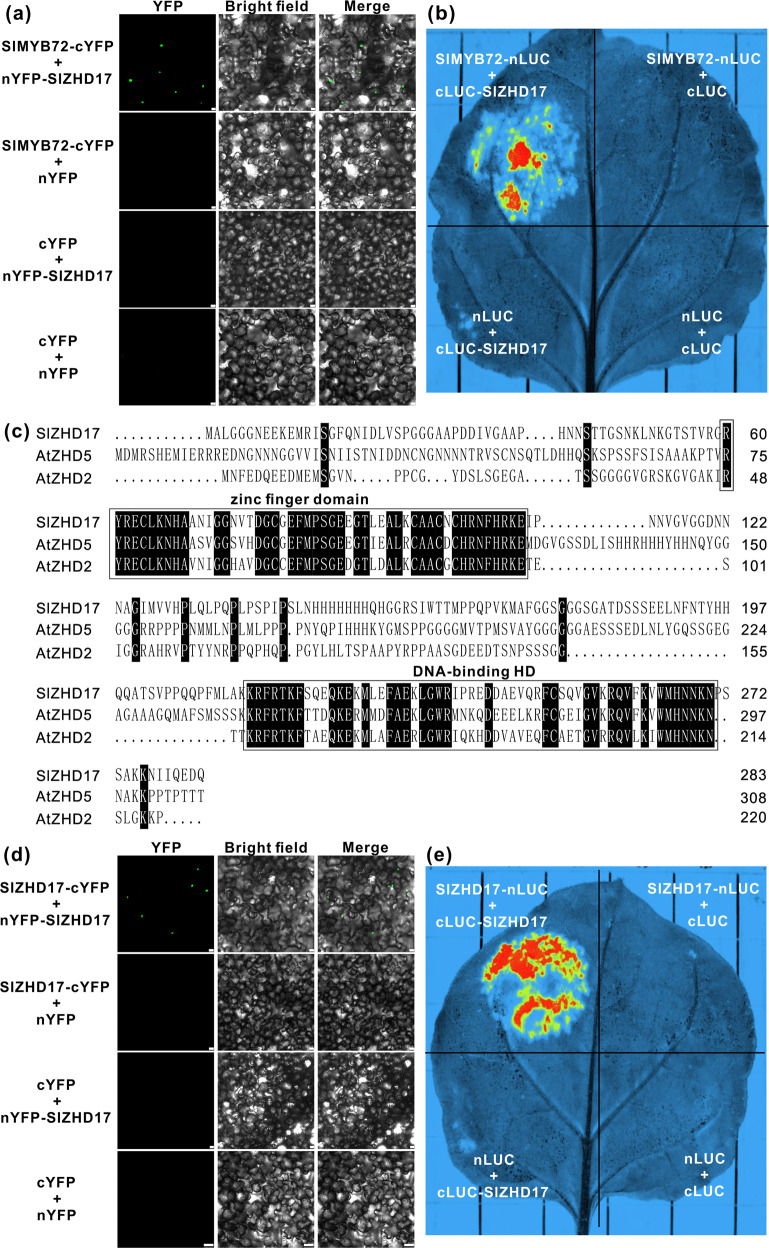
SlMYB72 interacts with the zinc-finger homeodomain transcription factor SlZHD17. **a** Protein–protein interaction between SlMYB72 and SlZHD17 in tobacco (*Nicotiana benthamiana*) leaves by bimolecular fluorescent complementation (BiFC) assay. Bar = 25 μm. **b** Interaction between SlMYB72 and the SlZHD17 protein in tobacco leaves by firefly luciferase complementation imaging (LCI) assay. **c** Sequence alignment analysis of SlZHD17, AtZHD2 (also named AtHB-22 and AT4G24660), and AtZHD5 (also named AtHB-33 and AT1G75240). White letters with a black background indicate identical amino acids, and the highly conserved zinc-finger domain and the DNA-binding homeodomain are boxed. The detailed amino acid sequences are provided in Appendices S2 and S3. **d** SlZHD17 homodimerization based on BiFC assay in tobacco leaves. Bar = 25 μm. **e** SlZHD17 forms a homodimer as assessed by LCI assay

We have shown the important role of SlMYB72 in the regulation of chlorophyll and carotenoid biosynthesis in a previous study^26^. Here, the SlMYB72-interacting protein SlZHD17 was identified. We verified the interaction by bimolecular fluorescent complementation assay (BiFC) and firefly luciferase complementation imaging assay (LCI) in tobacco (*Nicotiana benthamiana*) leaves (Fig. 1a, b), suggesting that SlZHD17 may play a role during tomato fruit development and ripening similar to SlMYB72.

There are 22 *SlZHD* genes in the tomato (*Solanum lycopersicum*) genome, and a phylogenetic relationship analysis of Arabidopsis (*Arabidopsis thaliana*), rice (*Oryza sativa*), and tomato ZHDs indicated that SlZHD17 belongs to clade III and has a close phylogenetic relationship with AtZHD5^25^. The open reading frame (ORF) of the *SlZHD17* gene in the Solanaceae Genomics Network (SGN) database is 873 bp. When the full-length cDNA of tomato fruit (cv. Micro-Tom) was amplified, an 855 bp ORF was identified that encodes 285 amino acids (Appendix S1, 2). JASPAR^2020^ is an open-access database of transcription factor binding profiles, and the recognition and binding motif sequences of AtZHD5 (AtHB-33), AtZHD2 (AtHB-22), and AtZHD3 (AtHB-21) have been provided in the database. Amino acid sequence alignment analysis of SlZHD17, AtZHD5, AtZHD2, and AtZHD3 showed that the ZF domain and HD domain are highly conserved in SlZHD17, AtZHD5, and AtZHD2 (Figs. 1c, S1, Appendix S3), suggesting that SlZHD17 may recognize the same binding motif. AtZHD3 was not listed here because the conserved domain sequences were quite different from the other three genes (Fig. S1). The positions of the conserved ZF domain and HD domain were at amino acids 60–112 and 213–269, respectively (Fig. 1c, Appendix S2). It has been reported that the ZF domain is crucial for dimerization^27^. Here, we also confirmed that SlZHD17 can form a homodimer by BiFC (Fig. 1d) and LCI assays (Fig. 1e).

### Downregulation of *SlZHD17* affects multiple agronomic traits in tomato plants

**Fig. 2:**
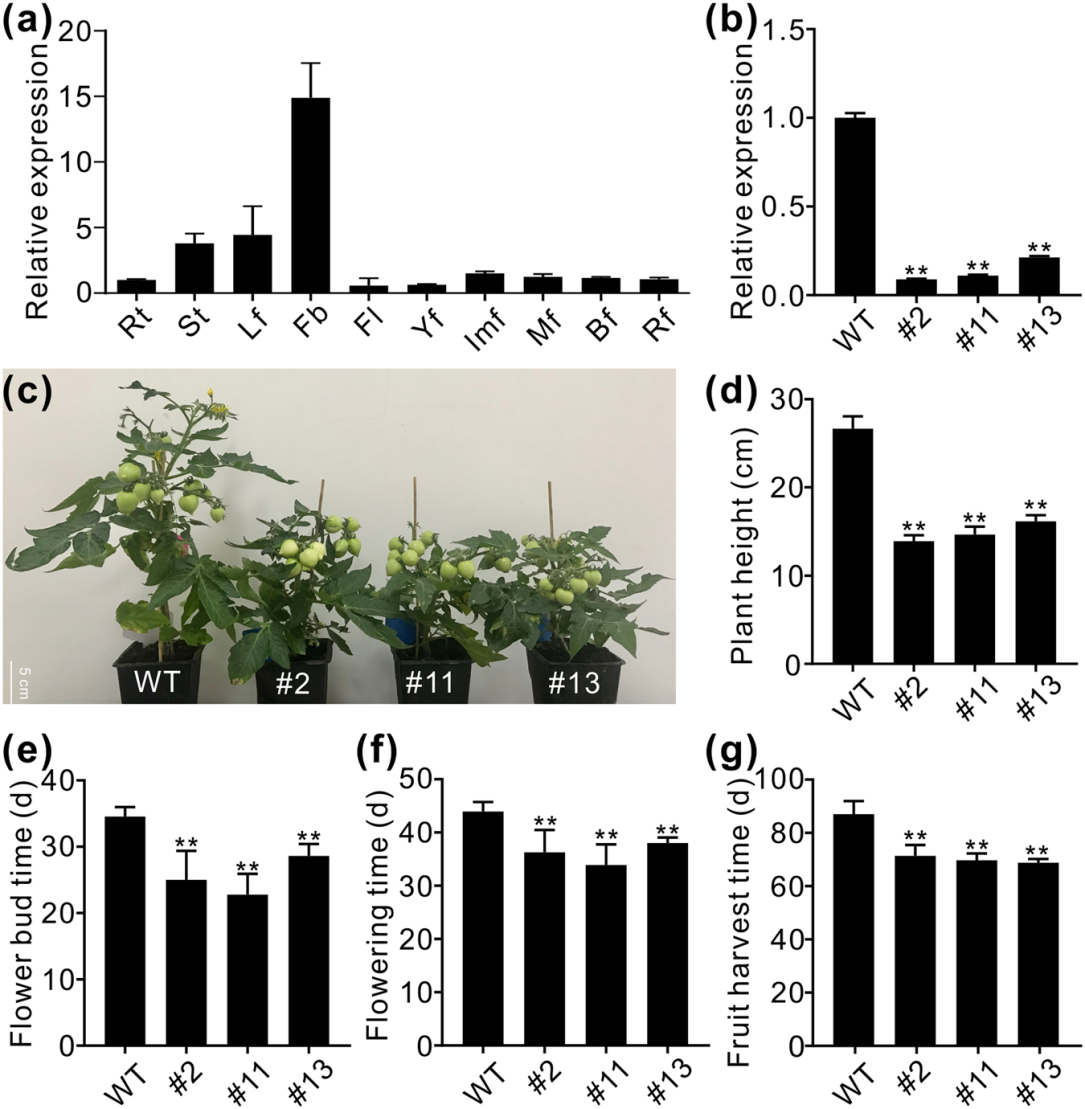
Tissue expression pattern of *SlZHD17* and phenotypic characterization of *SlZHD17*-RNAi plants. **a** Tissue expression pattern of the *SlZHD17* gene in WT plants. Rt root, St stem, Lf leaf, Fb flower bud, Fl flower, Yf young fruit, Imf immature green fruit, Mf mature green fruit, Bf breaker fruit, and Rf red ripe fruit. **b** The relative expression level of the *SlZHD17* gene in WT and T2 generation RNAi leaves. One-month-old plants were used for sampling and testing. The transcript level in WT was set as 1. Data represent the mean values of three independent experiments and error bars show the ±standard error values. Double asterisks (**) refer to significant differences between WT and transgenic lines with *P* < 0.01 (two-tailed Student’s *t*-test). **c** Photograph of WT and *SlZHD17*-RNAi plants at the fruit development stage. **d**–**g** Phenotypic characterization, including plant height (**d**), time of flower bud appearance (**e**), flowering time (**f**), and fruit harvest time (**g**), of WT and RNAi plants. The fruit harvest time in **g** was days from seedling transplant into soil to fruit ripening. In **d**–**g**, data represent the means of at least 12 values, and error bars show the standard error values; Double asterisks (**) refer to significant differences between WT and transgenic lines with *P* < 0.01 (two-tailed Student’s *t*-test)

Expression pattern analysis of the *SlZHD17* gene was performed in wild-type tomato. The relative expression level of *SlZHD17* in flower buds was much higher than that in other tissues, and relatively stable expression was found in fruits at different stages (Fig. 2a). For further functional study, *SlZHD17*-silenced transgenic plants were generated by RNA interference, and 14 independent transgenic lines were obtained and showed different degrees of suppression of *SlZHD17* (Fig. S2). Three lines with the most downregulated levels of *SlZHD17* (hereinafter referred to as #2, #11, and #13) were used for further experiments (Figs. 2b, S2). The *SlZHD17*-RNAi plants exhibited an obvious dwarf phenotype, and the plant height was significantly lower than that of the wild-type plants (Fig. 2c, d), but fruit set was not influenced. When flower buds appeared, the flowering time and fruit harvesting time of *SlZHD17*-RNAi plants were all significantly earlier than those of WT plants (Fig. 2e–g). These phenotypes indicated that downregulation of *SlZHD17* affects multiple agronomic characteristics in tomato plants, and *SlZHD17* is important for normal growth and development.

### SlZHD17 negatively regulates chlorophyll accumulation and chloroplast development in tomato fruit

**Fig. 3:**
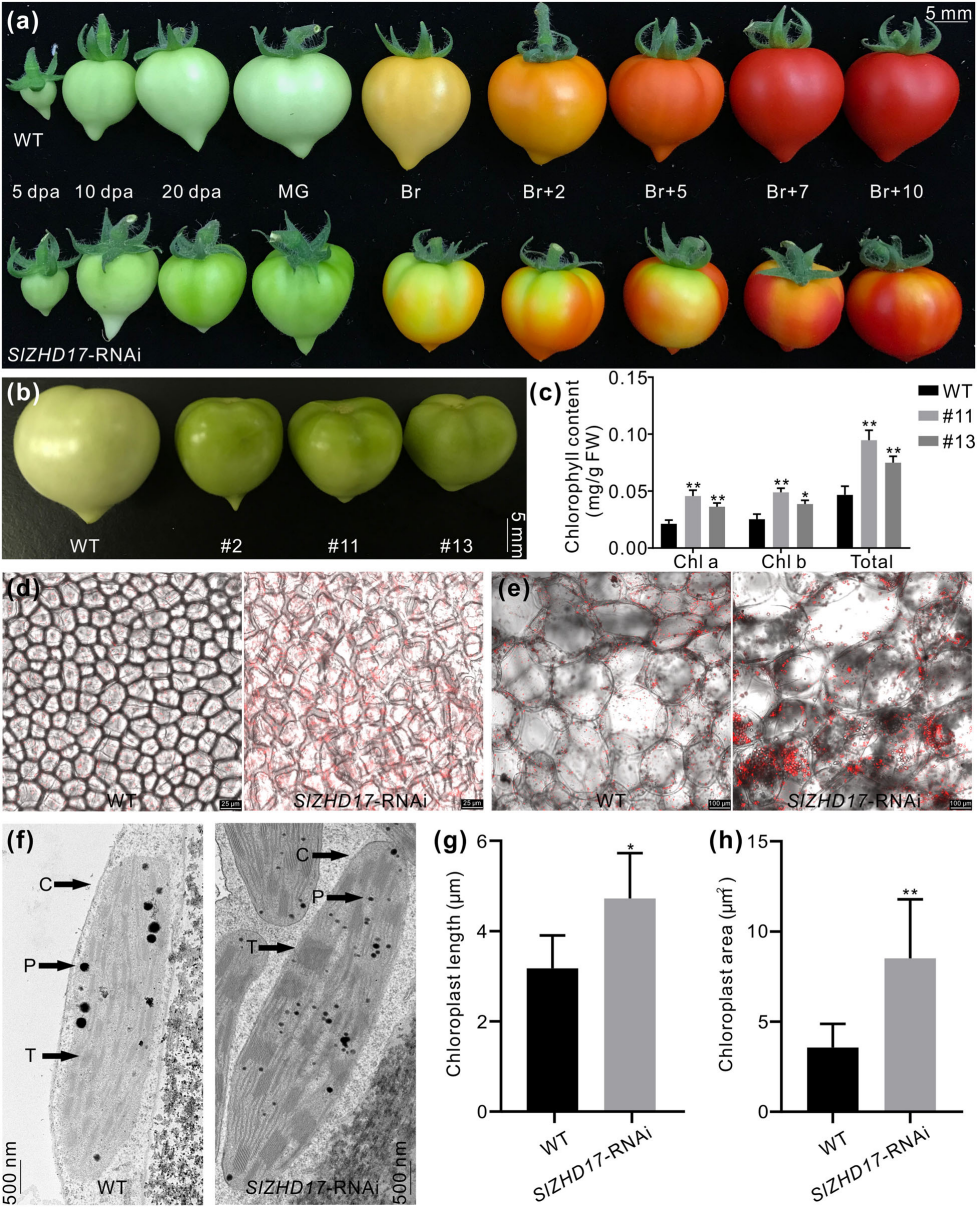
SlZHD17 regulates chlorophyll accumulation and chloroplast development in mature green fruit. **a** Photograph of WT and *SlZHD17*-RNAi (line #11) fruit at different stages. Dpa days post anthesis, MG mature green stage, Br breaker stage, and Br+2, Br+5, Br+7, and Br+10 indicate 2 days, 5 days, 7 days, and 10 days post breaker stage, respectively. **b** Photograph of WT and *SlZHD17*-RNAi fruit at the mature green stage. **c** The contents of chlorophyll a, chlorophyll b, and total chlorophyll in WT and *SlZHD17*-RNAi fruit at the mature green stage. FW fresh weight. Data represent the mean values of three independent experiments, and error bars show the standard error values. Single asterisk (*) and double asterisks (**) refer to significant differences between WT and transgenic lines with *P* < 0.05 and *P* < 0.01, respectively (two-tailed Student’s *t*-test). **d**, **e** Chlorophyll autofluorescence in the exocarp (**d**) and mesocarp (**e**) of WT and *SlZHD17*-RNAi (line #11) mature green fruit observed by confocal laser scanning microscopy. **f** Chloroplast ultrastructure in the mesocarp of WT and *SlZHD17*-RNAi (line #11) mature green fruit observed by transmission electron microscopy (TEM). C chloroplasts, P plastoglobules, and T thylakoid grana stacks. **g**, **h** Chloroplast length (**g**) and chloroplast area (**h**) of WT and *SlZHD17*-RNAi (line #11) mature green fruit. Data represent the mean values (WT, *n* = 5; *SlZHD17*-RNAi, *n* = 9), and error bars show the standard error values. Single asterisk (*) and double asterisks (**) refer to significant differences between WT and transgenic lines with *P* < 0.05 and *P* < 0.01, respectively (two-tailed Student’s *t*-test)

The transgenic fruit appeared much greener than the WT fruit at the immature and mature green stages and exhibited very obviously uneven pigmentation when the fruit entered the ripening stages (Figs. 3a, b, S3). Higher chlorophyll contents were found in *SlZHD17*-RNAi fruit than in WT fruit at the mature green stage (Fig. 3c). Stronger chlorophyll autofluorescence in the exocarp (Fig. 3d) and mesocarp (Fig. 3e) in RNAi fruit was observed by confocal laser scanning microscopy, which further verified greater chlorophyll accumulation in *SlZHD17*-RNAi fruit. Furthermore, the chloroplast ultrastructure in the mesocarp of WT and *SlZHD17*-RNAi mature green fruit was observed by transmission electron microscopy. The chloroplasts of RNAi fruit were significantly larger than those in WT fruit, and more thylakoid grana stacks were found in RNAi chloroplasts (Fig. 3f–h). These results indicated that downregulation of the *SlZHD17* gene influences chloroplast development and enhances chlorophyll accumulation in tomato fruit.

### Downregulation of *SlZHD17* alters the expression of genes involved in photosynthesis and carbohydrate metabolism

To further clarify the function of SlZHD17, RNA-seq of WT, and *SlZHD17*-RNAi fruit at the mature green stage was performed. There were 3821 differentially expressed genes (DEGs) between WT and RNAi fruit; 2839 DEGs were upregulated and 982 DEGs were downregulated, suggesting that SlZHD17 may have a primary function in transcriptional suppression at this stage (Fig. S4a, Data S1). Gene Ontology (GO) enrichment analysis of the DEGs showed that SlZHD17 mainly regulates MF (molecular function) and BP (biological process) pathways (Fig. S4b, Data S2). The most significantly enriched pathways included catalytic activity (GO:0003824); hydrolase activity, hydrolyzing O-glycosyl compounds (GO:0004553); photosynthesis, light harvesting (GO:0009765); carbohydrate metabolic process (GO:0005975) and cell wall organization or biogenesis (GO:0071554) (Fig. S5, Data S2). Kyoto Encyclopedia of Genes and Genomes (KEGG) enrichment analysis showed that the upregulated DEGs were mainly involved in photosynthesis, phenylpropanoid biosynthesis, and starch and sucrose metabolism pathways (Fig. S4c, Data S3), and the downregulated DEGs were mainly involved in protein processing in the endoplasmic reticulum and plant hormone signal transduction (Fig. S4d, Data S3). These results showed that downregulation of *SlZHD17* mainly affects photosynthesis and carbohydrate metabolism in tomato fruit.

### SlZHD17 influences chlorophyll biosynthesis and chloroplast development by directly regulating the *SlPOR-B* and *SlTKN2* genes

**Fig. 4:**
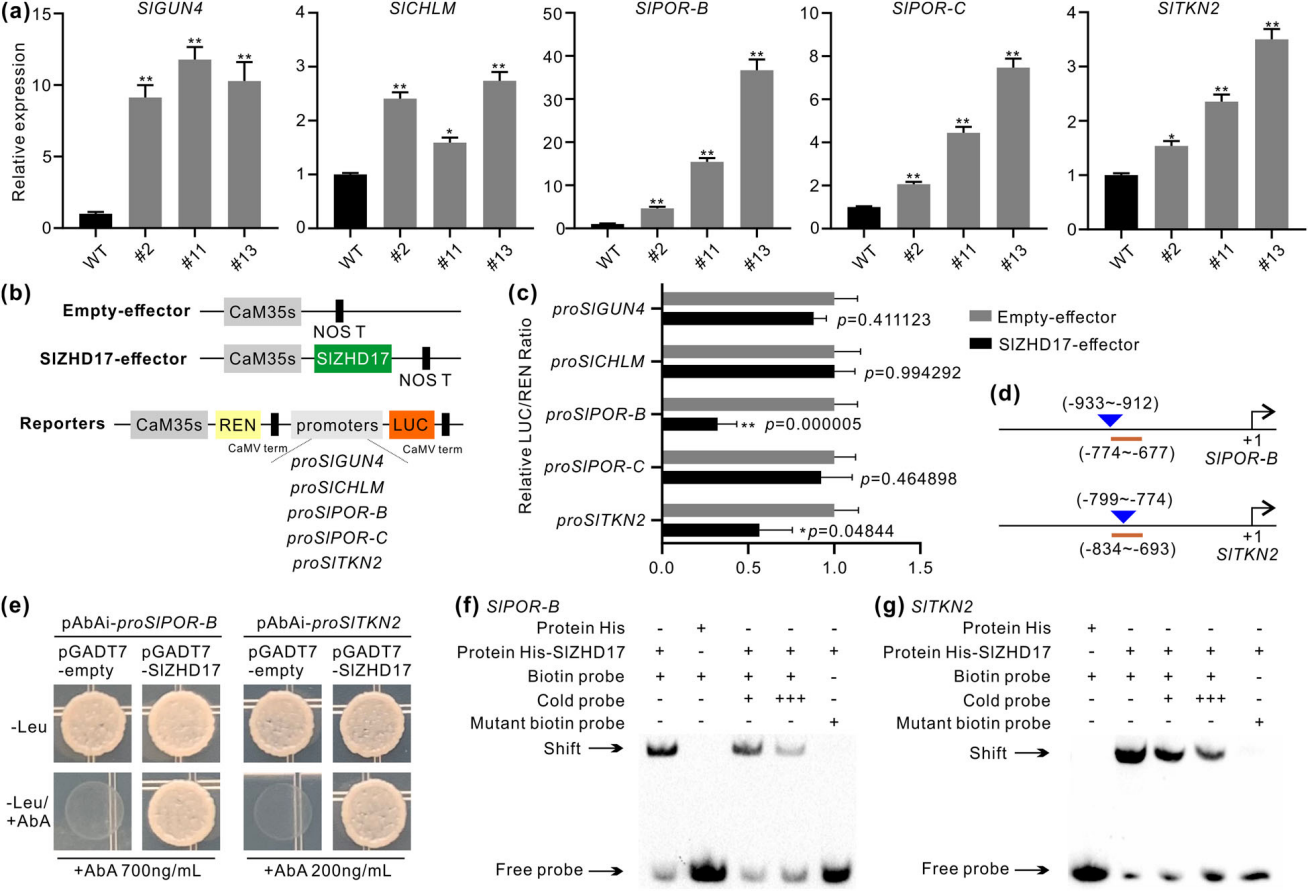
SlZHD17 directly regulates *SlPOR-B* and *SlTKN2* genes. **a** The relative expression levels of *SlGUN4*, *SlCHLM*, *SlPOR-B*, *SlPOR-C*, and *SlTKN2* in WT and *SlZHD17*-RNAi fruit at the mature green stage. The transcript level in WT was set as 1. Data represent the mean values of three independent experiments, and error bars show the standard error values. Single asterisk (*) and double asterisks (**) refer to significant differences between WT and transgenic lines with *P* < 0.05 and *P* < 0.01, respectively (two-tailed Student’s *t*-test). **b** Structural schematic diagrams of the effector and reporter plasmids used for the dual-luciferase assay. REN *Renilla luciferase,* LUC *firefly luciferase*. **c** Regulation of the *SlGUN4*, *SlCHLM*, *SlPOR-B*, *SlPOR-C,* and *SlTKN2* gene promoters by SlZHD17 based on dual-luciferase assay. The empty effector was used as a control (set as 1). Data represent the mean values of six independent experiments, and error bars show the standard error values. Single asterisk (*) and double asterisks (**) refer to significant differences between empty effector and SlZHD17-effector with *P* < 0.05 and *P* < 0.01, respectively (two-tailed Student’s *t*-test). The *P* values are provided in the graph. **d** Schematic diagrams of sequence positions chosen for yeast one-hybrid assay and electrophoretic mobility shift assay (EMSA). The underlined region indicates the promoter fragment used for the yeast one-hybrid assay, and the triangle indicates the sequence position used for the EMSA. The detailed sequences are provided in Appendix S4. **e** SlZHD17 binding with *SlPOR-B* and *SlTKN2* promoter fragments assessed by yeast one-hybrid assay. The yeast transformants were cultured on SD/−Leu and SD/−Leu/+AbA media for 3–5 days, and the pGADT7-empty plasmid was used as a control. **f**, **g** SlZHD17 binding with *SlPOR-B* (**f**) and *SlTKN2* (**g**) promoter regions in vitro by EMSA. TF-His protein incubated with a biotin-labeled DNA probe was used as a negative control. ‘+++’ indicates an increase in the competitor probe; no shift in the band for the His-SlZHD17 protein and mutant biotin-labeled DNA probe further confirmed the specific binding site

We further checked the relative expression levels of genes involved in chlorophyll biosynthesis and chloroplast development in WT and *SlZHD17*-RNAi fruit at the mature green stage. The chlorophyll biosynthesis genes *SlGUN4*, *SlCHLM*, *SlPOR-B*, and *SlPOR-C* were expressed at significantly higher levels in RNAi fruit than in WT fruit (Fig. 4a), whereas the expression of other genes, including *SlHEMA1*, *SlALAD*, *SlCHLH*, *SlCHLI*, *SlCHLD*, *SlPOR-A*, *SlCAO1,* and *SlCAO2*, was not influenced or was downregulated in RNAi fruit (Fig. S6a). The expression level of the chloroplast development gene *SlTKN2* was also significantly higher in RNAi fruit (Fig. 4a), whereas *SlTKN4* was not changed (Fig. S6b). A dual-luciferase assay was performed to detect whether SlZHD17 could directly regulate the promoter activity of *SlGUN4*, *SlCHLM*, *SlPOR-B*, *SlPOR-C*, and *SlTKN2*. The results showed that only the *SlPOR-B* and *SlTKN2* promoters were significantly suppressed by SlZHD17 (Fig. 4b, c). It has been reported that the core conserved binding motif of the ZHD transcription factor is ATTA/TAAT^20^, which is consistent with the binding motif of AtZHD5 and AtZHD2 in JASPAR^2020^ (Table S1). Multiple ZHD binding motifs in the *SlPOR-B* and *SlTKN2* promoters were predicted in JASPAR^2020^ (Appendix S4). Therefore, yeast one-hybrid assays and electrophoretic mobility shift assays (EMSAs) were further performed to confirm that SlZHD17 directly binds to the promoter regions of the *SlPOR-B* and *SlTKN2* genes (Fig. 4d–g). These results indicated that SlZHD17 modulates chlorophyll biosynthesis and chloroplast development by regulating the *SlPOR-B* and *SlTKN2* genes in a direct manner.

### SlZHD17 disturbs chlorophyll degradation by regulating *SlSGR1* in a direct manner

**Fig. 5:**
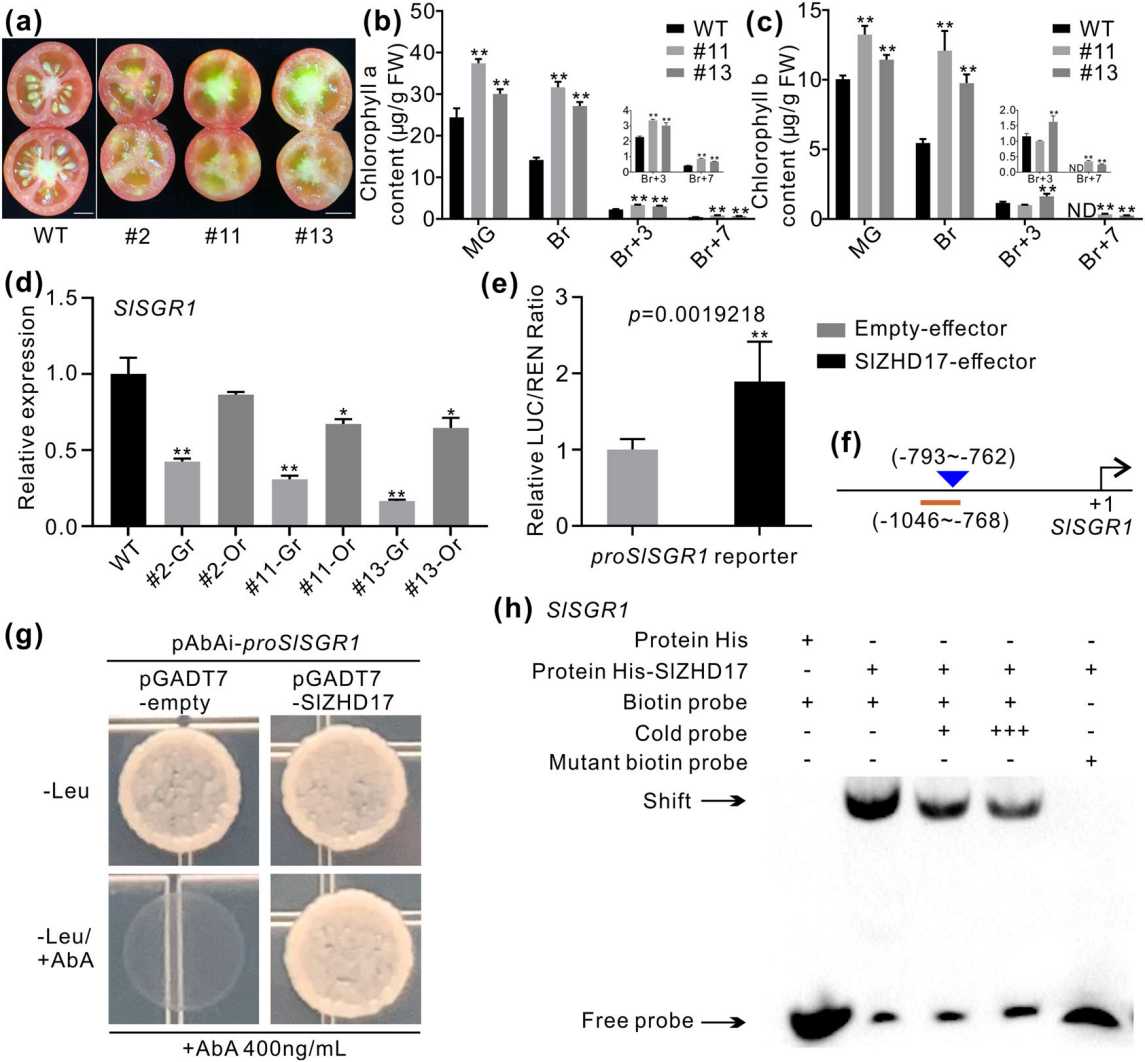
Inhibition of chlorophyll degradation in *SlZHD17*-RNAi fruit by directly regulating *SlSGR1*. **a** Photograph of the cross section of WT and *SlZHD17*-RNAi fruit at the Br+3 stage. Bar = 5 mm. **b**, **c** The contents of chlorophyll a (**b**) and chlorophyll b (**c**) in WT and *SlZHD17*-RNAi fruit at different stages detected by high-performance liquid chromatography (HPLC). FW fresh weight, ND not detected. **d** The relative expression level of *SlSGR1* in WT and *SlZHD17*-RNAi Br+3 fruit. For each transgenic fruit, the normal orange part (Or) and abnormal green part (Gr) were sampled separately. In **b**–**d**, data represent the mean values of three independent experiments, and error bars show the standard error values. Single asterisk (*) and double asterisks (**) refer to significant differences between WT and transgenic lines with *P* < 0.05 and *P* < 0.01, respectively (two-tailed Student’s *t*-test). **e** SlZHD17 activates the *SlSGR1* gene promoter, as determined by dual-luciferase assay. The empty effector was used as a control (set as 1). Data represent the mean values of six independent experiments, and error bars show the standard error values. Double asterisks (**) refer to significant difference between empty effector and SlZHD17-effector with *P* < 0.01 (two-tailed Student’s *t*-test). The *P* value is provided in the graph. **f** Schematic diagram of the sequence positions chosen for yeast one-hybrid assay and electrophoretic mobility shift assay (EMSA). The underlined region indicates the promoter fragment used for the yeast one-hybrid assay, and the triangle indicates the sequence position used for the EMSA. The detailed sequences are provided in Appendix S4. **g** SlZHD17 binding with the *SlSGR1* promoter fragment assessed by yeast one-hybrid assay. The yeast transformants were cultured on SD/−Leu and SD/−Leu/+AbA media for 3–5 days, and the pGADT7-empty plasmid was used as a control. **h** SlZHD17 binding to the *SlSGR1* promoter region in vitro by EMSA. TF-His protein incubated with biotin-labeled DNA probe was used as a negative control. ‘+++’ indicates an increase in the competitor probe; no shift in the band for the His-SlZHD17 protein and mutant biotin-labeled DNA probe further confirmed the specific binding site

Chlorophyll was degraded during the fruit ripening process, which was accompanied by carotenoid accumulation. The pericarp, septum, placenta, and locular tissue of wild-type fruit at the Br+3 stage appeared orange to pink, and the columella was usually white (Fig. 5a). However, parts of the pericarp, placenta and locular tissue of *SlZHD17*-RNAi fruit at the Br+3 stage were still green (Fig. 5a). The detailed contents of chlorophyll a and chlorophyll b at the mature green, breaker, Br+3, and Br+7 stages in WT and *SlZHD17*-RNAi fruit were measured by high-performance liquid chromatography (HPLC). Chlorophyll a and b in WT fruit were both significantly reduced from the mature green stage to the breaker stage, and they were lower at the Br+3 stage but basically absent at the Br+7 stage (Fig. 5b, c). In contrast to WT fruit, the chlorophyll contents in *SlZHD17*-RNAi fruit were much higher than those in WT fruit at each stage, and there were no significant decreases from the mature green stage to the breaker stage (Fig. 5b, c). These phenotypes indicated that the degradation of chlorophyll in *SlZHD17*-RNAi fruit was disturbed.

It has been clarified that *SlSGR1* encodes a STAYGREEN protein that plays an important role in the regulation of chlorophyll degradation^28,29^. Silencing of *SlSGR1* leads to reduced chlorophyll degradation in tomato leaves and fruit^30^. We divided the uneven pigmentation RNAi fruit into two parts, including the normal orange part (Or) and abnormal green part (Gr). The relative expression level of *SlSGR1* was significantly lower in both the orange part and green part of *SlZHD17*-RNAi fruit than in WT fruit at the Br+3 stage (Fig. 5d). To confirm whether the disturbed chlorophyll degradation in *SlZHD17*-RNAi fruit was caused by the suppression of *SlSGR1*, a dual-luciferase assay was performed and showed that SlZHD17 activates the *SlSGR1* promoter in tobacco leaves (Fig. 5e). Yeast one-hybrid assays and EMSAs further verified that SlZHD17 binds to the *SlSGR1* promoter fragment (Fig. 5f–h). These results suggested that SlZHD17 influences chlorophyll degradation by directly regulating *SlSGR1*.

### SlZHD17 affects the plastid transition and carotenoid accumulation in tomato fruit

**Fig. 6:**
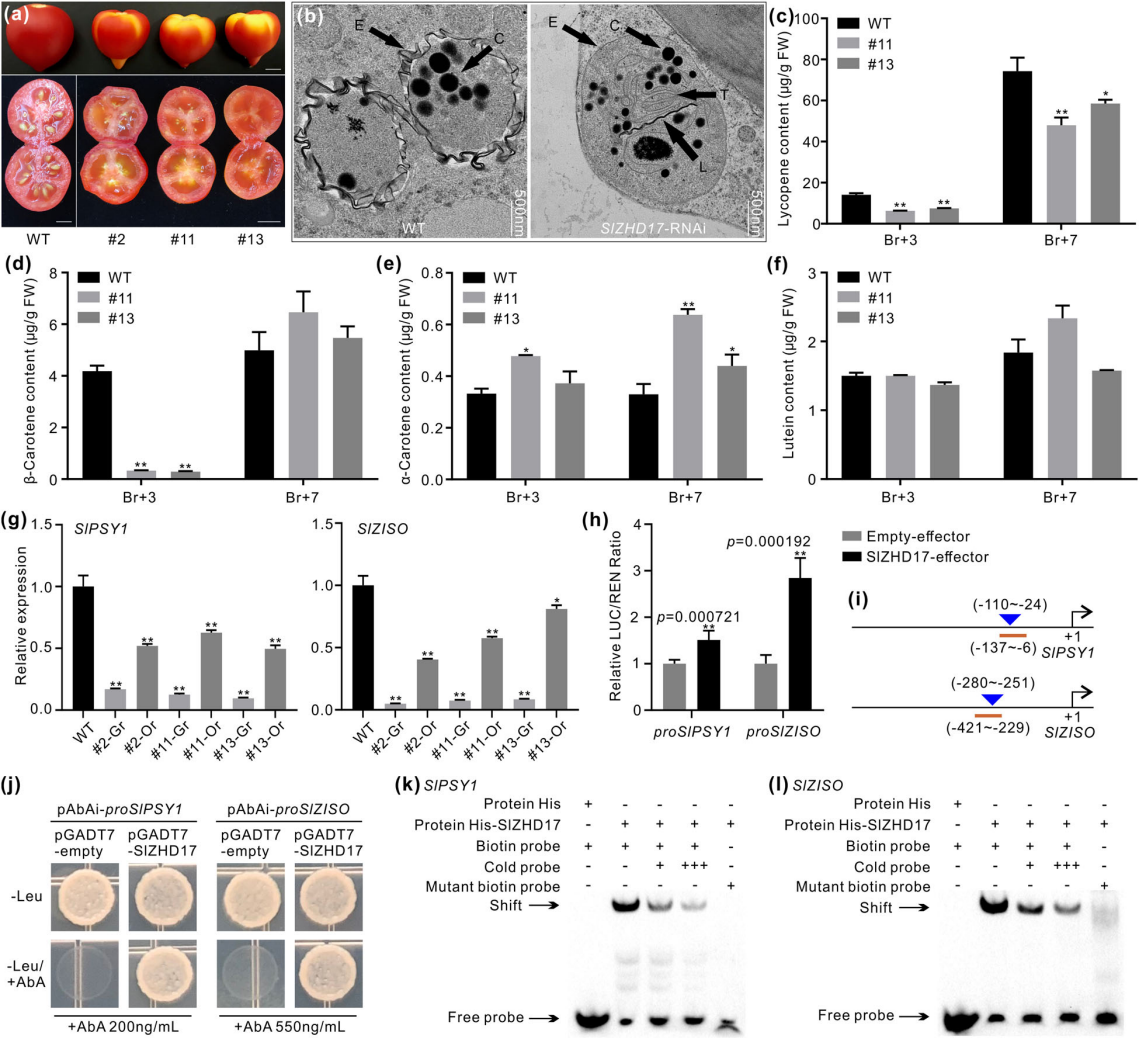
SlZHD17 influences carotenoid accumulation by directly regulating the *SlPSY1* and *SlZISO* genes. **a** Photograph of the whole and cross sections of WT and *SlZHD17*-RNAi fruit at the Br+7 stage. Bar = 5 mm. **b** Ultrastructure of chromoplasts in the mesocarp of WT and *SlZHD17*-RNAi (line #11) Br+3 fruit observed by TEM. C carotenoid-containing globules, E plastid envelope, L crystal line, and T thylakoid grana. **c**–**f** The content of lycopene (**c**), β-carotene (**d**), α-carotene (**e**), and lutein (**f**) in WT and *SlZHD17*-RNAi fruit at the Br+3 and Br+7 stages by HPLC. FW, fresh weight. **g** The relative expression levels of *SlPSY1* and *SlZISO* in WT and *SlZHD17*-RNAi Br+3 fruit. For each transgenic fruit, the normal orange part (Or) and abnormal green part (Gr) were sampled separately. In **c**–**g**, data represent the mean values of three independent experiments, and error bars show the standard error values. Single asterisk (*) and double asterisks (**) refer to significant differences between WT and transgenic lines with *P* < 0.05 and *P* < 0.01, respectively (two-tailed Student’s *t*-test). **h** Regulation of SlZHD17 on *SlPSY1* and *SlZISO* gene promoters based on dual-luciferase assay. The empty effector was used as a control (set as 1). Data represent the mean values of six independent experiments, and error bars show the standard error values. Double asterisks (**) refers to significant differences between empty effector and SlZHD17-effector with *P* < 0.01, respectively (two-tailed Student’s *t*-test). The *P* values were provided in the graph. **i** Schematic diagrams of sequence positions chosen for yeast one-hybrid assay and electrophoretic mobility shift assay (EMSA). The underlined region indicates the promoter fragment used for the yeast one-hybrid assay, and the triangle indicates the sequence position used for the EMSA. The detailed sequences are provided in Appendix S4. **j** SlZHD17 binding with *SlPSY1* and *SlZISO* promoter fragments assessed by yeast one-hybrid assay. The yeast transformants were cultured on SD/−Leu and SD/−Leu/+AbA media for 3–5 days, and the pGADT7-empty plasmid was used as a control. **k**, **l** SlZHD17 binding with *SlPSY1* (**k**) and *SlZISO* (**l**) promoter regions in vitro by EMSA. TF-His protein incubated with a biotin-labeled DNA probe was used as a negative control. ‘+++’ indicates an increase in the competitor probe; no shift in the band for the His-SlZHD17 protein and mutant biotin-labeled DNA probe further confirmed the specific binding site

The uneven pigmentation of *SlZHD17*-RNAi fruit was still obvious at the Br+7 stage, and the previously green part at the Br+3 stage became an yellow/orange color. The cross section of the pericarp and inner tissues in WT fruit was completely red at the Br+7 stage, whereas a light red color was observed in *SlZHD17*-RNAi fruit (Fig. 6a), suggesting that in addition to the obviously uneven pigmentation, carotenoid accumulation was also changed. Chromoplasts are commonly derived from chloroplasts in fruits and vegetables that undergo green to yellow or red color changes during ripening^3^. This plastid differentiation process includes the breakdown of chloroplasts in the thylakoid membrane, the formation of chromoplasts in the inner membrane envelope, and the emergence of carotenoid-containing structures^31^. Therefore, we further observed the ultrastructure of chromoplasts in the equatorial region mesocarp of WT and *SlZHD17*-RNAi fruit at the Br+3 stage. Undulating membrane structures were observed in WT fruit, whereas the plastid envelope in RNAi fruit was still the chloroplast membrane. The carotenoid-containing globules in RNAi fruit were also smaller than those in WT fruit, and noticeable residues of thylakoid grana were found in RNAi fruit (Fig. 6b). These ultrastructure results suggested that chloroplast conversion into chromoplasts was delayed in *SlZHD17*-RNAi fruit.

Furthermore, the measurement of carotenoid components in WT and transgenic fruit at the Br+3 and Br+7 stages confirmed this phenotype. The red pigmentation of ripe tomato is due to lycopene, which accounts for 70–90% of the carotenoids in most varieties, while β-carotene accounts for the bulk of the remainder. Lutein is yellow and accounts for a minor fraction of normal ripe tomato carotenoids^31,32^. The lycopene contents were significantly lower in RNAi fruit than in WT fruit at both the Br+3 and Br+7 stages (Fig. 6c). The β-carotene content in RNAi fruit was lower than that in WT fruit at the Br+3 stage but then increased to the same level as that in WT fruit at the Br+7 stage (Fig. 6d). The α-carotene content in RNAi fruit was higher than that in WT fruit (Fig. 6e). There was no difference in the lutein content between WT and transgenic fruit (Fig. 6f). These results revealed that downregulation of *SlZHD17* disturbed chromoplast development and carotenoid accumulation in tomato fruit.

### SlZHD17 directly regulates *SlPSY1* and *SlZISO* genes

To further explore the mechanism by which SlZHD17 modulates carotenoid biosynthesis in tomato fruit, the expression of carotenoid biosynthesis genes was also examined. The relative expression levels of *SlPSY1* and *SlZISO* were significantly lower in both the orange part and green part of *SlZHD17*-RNAi fruit than in WT fruit at the Br+3 stage, and more importantly, the levels in the green part were much lower than those in the orange part (Fig. 6g). Other carotenoid biosynthesis genes, including *SlPDS*, *SlZDS*, *SlCRTISO*, and *SlCYC-B*, were also suppressed in the abnormal green part but not suppressed in the normal orange part, except that the *SlCYC-B* gene was suppressed in both parts (Fig. S7). In addition, the expression of *SlPSY1* in the fruit of the two *SlZHD17*-RNAi lines at the Br+7 stage was also much lower than that in the WT, while the other five genes were not significantly downregulated at the Br+7 stage (Fig. S8). The lower contents of lycopene and β-carotene in *SlZHD17*-RNAi fruit may be caused by the suppression of *SlPSY1* and *SlZISO*. The dual-luciferase assay confirmed that SlZHD17 directly activates the *SlPSY1* and *SlZISO* promoters (Fig. 6h). Yeast one-hybrid assays and EMSAs further verified that SlZHD17 binds with *SlPSY1* and *SlZISO* promoter fragments (Fig. 6i–l). These results suggested that SlZHD17 influences carotenoid accumulation in tomato fruit by regulating the *SlPSY1* and *SlZISO* genes in a direct manner.

### SlZHD17 interacts with SlARF4, SlBEL11, and SlTAGL1 proteins

**Fig. 7:**
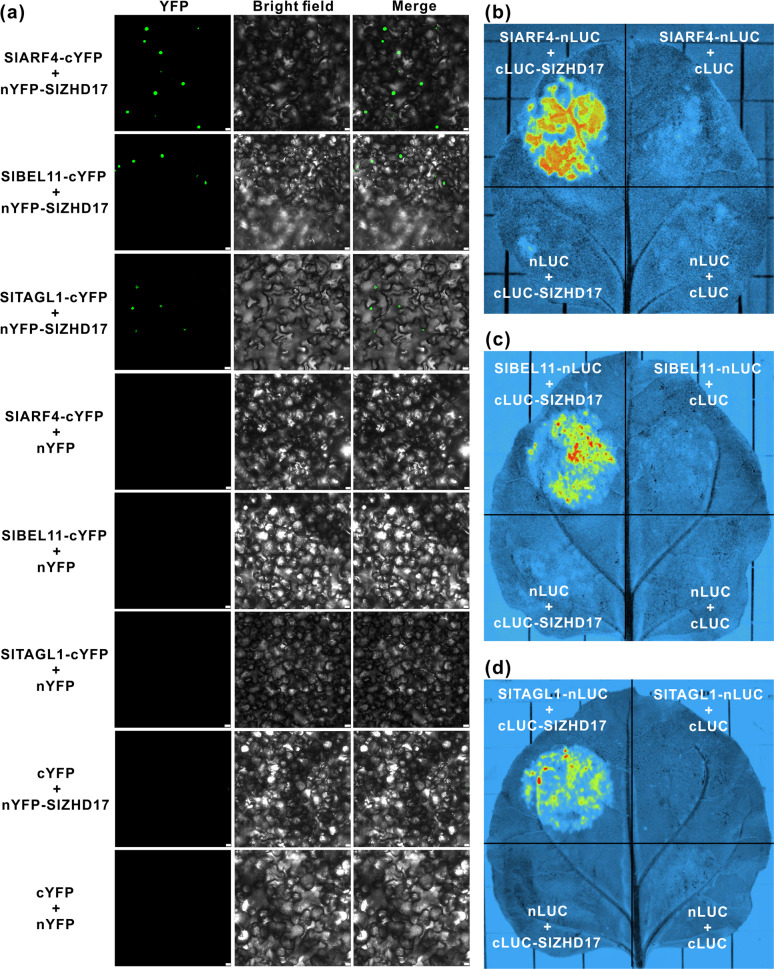
SlZHD17 interacts with the SlARF4, SlBEL11, and SlTAGL1 proteins. **a** Protein–protein interactions between SlZHD17 and the SlARF4, SlBEL11, and SlTAGL1 proteins in tobacco leaves by BiFC assay. Bar = 25 μm. **b**–**d** SlZHD17 interacts with SlARF4 (**b**), SlBEL11 (**c**), and SlTAGL1 (**d**) proteins in tobacco leaves by LCI assay

In previous studies, downregulation of *SlARF4*, *SlBEL11*, or *SlTAGL1* fruit exhibited phenotypes similar to those of *SlZHD17*-RNAi fruit^12,16,18,19^, suggesting that these transcription factors have similar regulatory functions in pigment metabolism. The protein–protein interactions between SlZHD17 and the SlARF4, SlBEL11, and SlTAGL1 proteins were observed in tobacco leaves by BiFC (Fig. 7a) and LCI (Fig. 7b–d) assays. These results suggested that SlZHD17 interacts with the SlARF4, SlBEL11, and SlTAGL1 proteins, which coordinate to regulate pigment metabolism in tomato fruit.

## Discussion

Chlorophylls and carotenoids are essential to plants and beneficial to human health. The chlorophyll content of immature fruits is usually correlated with nutrients and flavor at maturity, and increasing the chlorophyll content and chloroplast development of immature fruits can improve photosynthesis efficiency and lead to the accumulation of more starch, the carbohydrate that correlates with the sugar content in ripe fruits^33^. Carotenoids provide colors to ripe fruits, and lycopene and β-carotene are powerful antioxidants that are essential for human health^31,32^. Therefore, it is necessary to identify the regulatory network of these pigments.

ZHD proteins belong to the HD superfamily, and dimerization of HD proteins is one of the mechanisms that modulates target genes. There have been no reports on the involvement of the ZHD protein in regulating pigment accumulation to date. In this study, we showed that SlZHD17 forms a homodimer (Fig. 1d, e) and acts as a negative regulator of chlorophyll biosynthesis and chloroplast development in tomato fruit (Fig. 3). Compared to WT fruit, stronger chlorophyll fluorescence in *SlZHD17*-RNAi fruit was observed, which was consistent with the larger chloroplasts and higher number of thylakoids (Fig. 3). Plastid development and pigment accumulation are influenced by numerous environmental and genetic factors. Tomato *SlGLK1* and *SlGLK2* are functionally redundant in chloroplast development and distribution; *SlGLK1* is more important in leaves, and *SlGLK2* is predominant in fruit^34^. Overexpression of *SlGLK2* enhances chlorophyll levels by increasing the number of plastids and their size^33,34^. There was no influence of *SlGLK1* expression in *SlZHD17*-RNAi fruit at the mature green stage, but *SlGLK2* was much higher in only one transgenic line than in WT fruit (Fig. S6b). Moreover, considering that the cv. Micro-Tom bears the inactive *u* allele of *SlGLK2*^13^, the increased chloroplast development and chlorophyll accumulation in *SlZHD17*-RNAi fruit did not seem to be caused by *SlGLKs*.

The *POR* gene encodes a protochlorophyllide oxidoreductase, which plays a crucial role in the chlorophyll biosynthetic pathway^35^. Several transcription factors, including SlMYB72, SlBEL11 and SlBL4, could influence chlorophyll biosynthesis by directly regulating *SlPOR-B* and *SlTKN2*^16,17,26^. Here, we showed that SlZHD17 could directly regulate the *SlPOR-B* and *SlTKN2* genes (Fig. 4). Furthermore, protein–protein interactions between SlZHD17 and SlMYB72 and SlBEL11 were found (Figs. 1, 7), suggesting that SlZHD17 may be coupled with SlMYB72 and SlBEL11 to synergistically modulate chlorophyll biosynthesis and chloroplast development in tomato fruit. In addition, SlBEL11 also belongs to the HD superfamily^16^, and the interaction of SlZHD17 and SlBEL11 proteins provides more evidence of heterodimerization of HD proteins. We also performed interaction assays between SlZHD17 and the SlBL4 protein, but no interaction was observed. Compared to WT fruit, the relative expression level of *SlBL4* was lower in *SlZHD17*-RNAi fruit at the mature green stage (Fig. S6b), suggesting that SlBL4 may function downstream of SlZHD17. In a previous study, SlMYB72 was found to interact with SlARF4^26^. Here, we showed that SlZHD17 also interacted with SlARF4 (Fig. 7), suggesting that this protein complex may work together to regulate chloroplast development and chlorophyll biosynthesis. On the other hand, downregulation of either *CUL4*, *DDB1*, or *DET1* will stabilize the SlGLK2 protein, which leads to increased levels of chlorophyll and carotenoids in tomato fruit^10^. The expression of *SlCUL4* in *SlZHD17*-RNAi fruit was not influenced, but *SlDDB1* was downregulated in the two transgenic lines (Fig. S6b). Considering that *SlGLK2* was not influenced in these two transgenic lines at the transcriptional level, the changes in chloroplasts and chlorophyll in RNAi fruit were not caused by this ubiquitin ligase complex.

*SlPSY1* encodes a phytoene synthase, which is the rate-limited step during carotenoid biosynthesis in tomato fruits^36^. Silencing or knocking out of *SlPSY1* in fruits resulted in a serious carotenoid deficiency^37,38^, followed by a series of desaturation and isomerization reactions catalyzed by phytoene desaturase (PDS), ζ-carotene desaturase (ZDS), ζ-carotene isomerase (ZISO), and carotenoid isomerase (CRTISO). Cyclization of lycopene by lycopene ε-cyclase (LycE) and lycopene β-cyclase (LycB) produces α-carotene and β-carotene, respectively^39^. Here, we also confirmed that SlZHD17 directly regulates the expression of the *SlPSY1* and *SlZISO* genes (Fig. 6). The decreased lycopene content in *SlZHD17*-RNAi fruit was due to the high level of direct suppression of the *SlPSY1* and *SlZISO* genes (Fig. 6). Furthermore, the important fruit ripening regulator RIN, which corresponds to the mutant *rin*, fails to accumulate lycopene in its fruits^40^, and RIN directly interacts with the *SlPSY1* promoter^41^. The RIN-interacting protein SlTAGL1 has been reported^42^, and tomato fruit with *SlTAGL1* transcriptional suppression exhibited dark green color at the early developmental stage and inhibited carotenoid biosynthesis at the ripe stage^18,19^. A recent study reported that SlTAGL1 positively regulates *SlPSY1* expression^43^. We also identified that SlMYB72 directly regulates *SlPSY1* expression^26^. In this study, a protein–protein interaction between SlZHD17 and SlTAGL1 was found (Fig. 7). The strongly suppressed *SlPSY1* in RNAi fruit was directly regulated by SlZHD17, yet other *SlPSY1* regulators, including SlMYB72 or SlTAGL1, may also contribute to this phenomenon. However, *SlSGR1* is a positively regulated target of RIN too^41^, and the SlSGR1 protein directly interacts with the SlPSY1 protein to inhibit its activity^29^. Even though the transcriptional levels of *SlSGR1* (Fig. 5) and *SlPSY1* (Fig. 6) were both downregulated in the RNAi fruit, we hold the opinion that the decreased lycopene content in RNAi fruit was due to the complex regulatory network. Although the degradation of chlorophyll and the reprogramming of carotenoids co-occur at the onset of fruit ripening, these two events are not necessarily interdependent^28^. Thus, although the chlorophyll content was higher in RNAi immature fruit, the lycopene content was lower in ripe fruit.

**Fig. 8:**
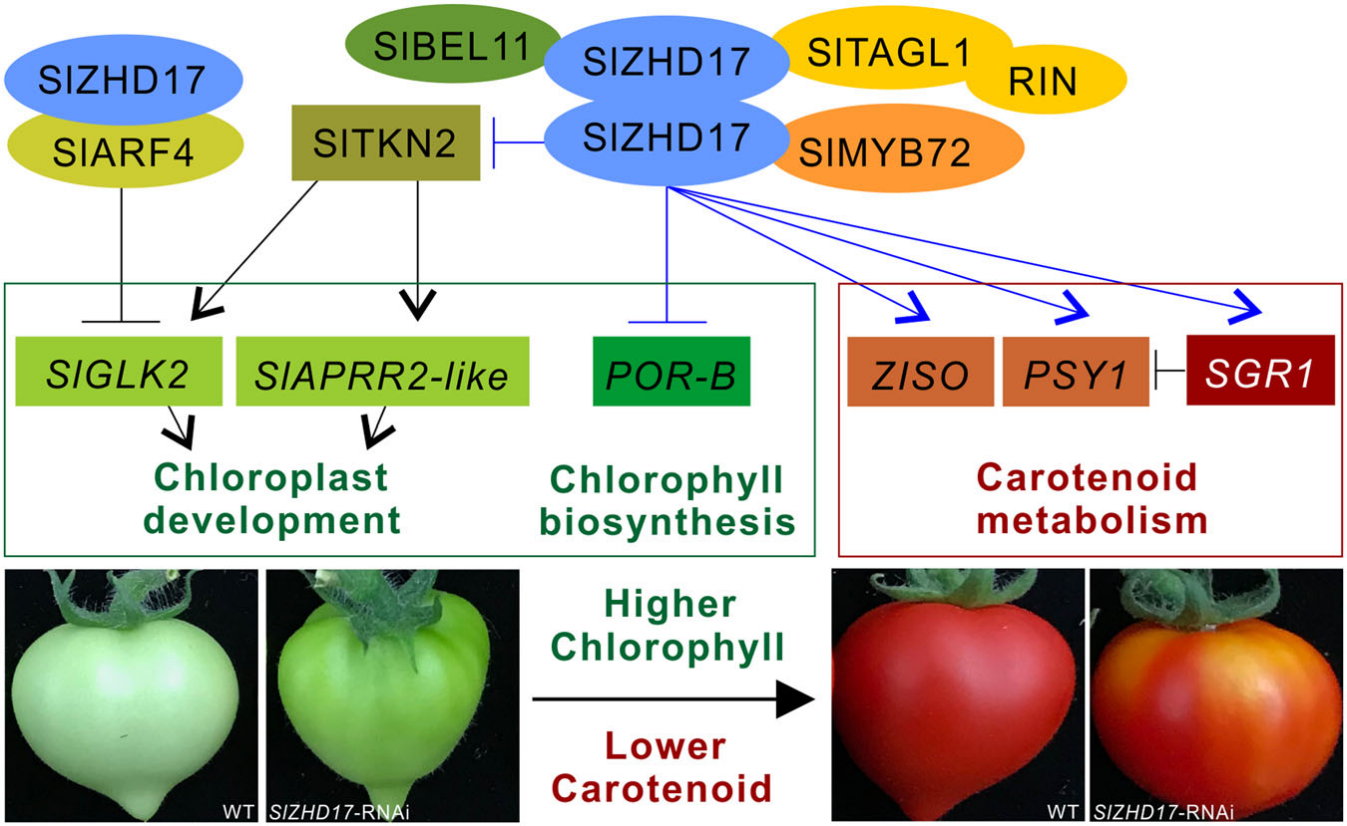
Proposed model for SlZHD17 regulation of pigment metabolism in tomato fruit. Our study uncovers a SlZHD17-regulated network involved in chlorophyll and carotenoid metabolism. The SlMYB72-interacting protein SlZHD17 directly regulates the *SlPOR-B* gene, which influences chlorophyll biosynthesis. SlZHD17 also regulates *SlTKN2* in a direct manner, which determines chloroplast development in tomato fruit. On the other hand, the important regulator of chlorophyll degradation, *SlSGR1*, and the crucial carotenoid biosynthesis genes *SlPSY1* and *SlZISO* are also directly regulated by SlZHD17. Furthermore, protein–protein interactions between SlZHD17 and other pigment regulators, including SlARF4, SlBEL11, and SlTAGL1, are also presented here. SlTAGL1 interacts with RIN, which is an important regulator of tomato fruit ripening. SlTKN2- and SlARF4-induced chloroplast development was referenced by Nadakuduti et al.^11^

In the silenced part of TRV-*SlBEL11* fruit, the dark-green region at the mature green stage could not turn red normally in Br+6 stage fruit^16^. The *SlARF4-*suppressed fruit also exhibited blotchy ripening^12^. An uneven color phenotype was also observed in *SlMYB72*-RNAi fruit^26^. In a recent study, high degrees of methylation of the *SlTAGL1* promoter resulted in suppressed expression, leading to green stripes on the fruit^43^. These SlZHD17-interacting proteins may form a protein complex that functions in both chlorophyll and carotenoid metabolism. The proposed model is summarized in Fig. 8. We also checked the relative expression level of the *SlZHD17* gene in the normal orange part and the abnormal green part in RNAi fruit. Compared to WT fruit, this gene was significantly downregulated in both parts but was much lower in the green part (Fig. S9), indicating that the uneven pigmentation may be caused by the different suppression levels of *SlZHD17*, which was similar to the results of other previous studies^26,43^.

In addition to participating in the regulation of pigment metabolism, downregulation of *SlZHD17* also induced dwarfism in plants, accelerated flowering, resulted in earlier fruit harvest, but had no influence on fruit set, which are beneficial agronomic traits (Fig. 2), suggesting that SlZHD17 has a broader function. Our study presents the function and mode of SlZHD17 involved in the control of chlorophyll and carotenoid metabolism in tomato fruit and provides new insight into the complex pigment regulatory network. These results also provide a new strategy for cultivating improved varieties of fleshy fruits.

## Materials and methods

### Sequence alignment

The sequence information of *SlZHD17* (Solyc04g080490) was downloaded from the Solanaceae Genomics Network (https://solgenomics.net/), and some differences in the ORF (open reading frame) were found when the full-length cDNA of tomato fruit (*Solanum lycopersicum* cv. Micro-Tom) was used as a template (Appendix S1). The amino acid sequences of AtZHD2 (also named AtHB-22 and AT4G24660), AtZHD3 (also named ATHB-21 and AT2G18550), and AtZHD5 (also named AtHB-33 and AT1G75240) were downloaded from UniProt (www.uniprot.org/uniprot/). Sequence alignment was performed by DNAMAN software.

### Plant materials and growth conditions

To generate RNA interference (RNAi) plants, a 259 bp sequence fragment (Appendix S1) amplified by PCR was cloned into the modified plant binary vector pCambia 1301 under the CaMV 35 S promoter^44^. The recombinant *SlZHD17*-RNAi plasmid was transformed into *Agrobacterium tumefaciens* strain GV3101, and *A. tumefaciens*-mediated infection methods were performed in wild-type tomato plants (*Solanum lycopersicum* cv. Micro-Tom). Kanamycin (100 mg/L) selection and PCR confirmation were used to screen positive transgenic lines, and the relative expression level of *SlZHD17* was confirmed by qRT–PCR. T2 or T3 generations were used for experiments, and all plants were grown in a greenhouse under controlled conditions (18 h light, 25 °C; 6 h dark, 18 °C; 60% relative humidity).

### Measurement of chlorophyll and carotenoid contents

The total chlorophyll content of WT and *SlZHD17*-RNAi mature green fruit measured by a spectrophotometric method was similar to that in our previous study^45^. Chlorophyll (chlorophyll a and chlorophyll b) and carotenoid (lycopene, β-carotene, α-carotene and lutein) contents were measured using HPLC methods. In brief, a 20 mg frozen fruit tissue sample was extracted with 1 mL cold hexane:acetone:methanol (2:1:1, v-v) solution for 15 min; then, 100 μL water was added for 20 s, and the sample was centrifuged at maximum speed for 3 min. The upper organic phase was dried by vacuum centrifugation followed by 150 μL ethyl acetate addition, and all the samples were filtered through a 0.22 μm syringe filter. HPLC analysis was performed according to Wu et al.^26^; an Agilent 1260 Series liquid chromatograph system (Agilent, USA) and a YMC C30 column (4.6 × 250 mm) were also used in this study. Quantification of individual substances was made by comparison with the peak areas of standard substances, and all experiments were performed with three independent biological replicates.

### Chlorophyll autofluorescence and plastid observations

For chlorophyll autofluorescence observations, the exocarp and mesocarp of WT and *SlZHD17*-RNAi mature green fruit were thinly sliced with a sharp blade and observed by confocal laser scanning microscopy (Leica, Germany). The emitted fluorescence of chloroplasts was collected at 650–750 nm. For chloroplast and chromoplast observations, the mesocarps of mature green fruit and Br+3 fruit were thinly sliced with a sharp blade and fixed in glutaraldehyde (2.5%) solution. The observations were performed with a Tecnai T12 TWIN transmission electron microscope (FEI, USA). The length and area of chloroplasts were calculated using ImageJ software.

### Expression analysis by qRT–PCR

Total RNA was extracted by an RNAprep Pure kit (TIANGEN, China), and first-strand cDNA synthesis and quantitative real-time PCR were performed using commercial kits (TAKARA, Japan). The Bio–Rad CFX system was used for the PCR procedure (Bio–Rad, USA). The relative expression levels of genes were calculated by the 2^−ΔΔCt^ method and normalized to the internal reference gene *SlActin*. Three biological replicates were performed for each sample. The qRT–PCR primers are listed in Table S2.

### RNA-seq analysis

WT and *SlZHD17*-RNAi (line #11) fruit at the mature green stage were harvested and used for RNA-seq analysis. Total RNA was extracted, and cDNA libraries were then constructed for sequencing by Shanghai Majorbio Biopharm Technology Co., Ltd. (China). Hisat2 was used to map clean reads to the reference genome of *Solanum lycopersicum* SL3.0 version (http://solgenomics.net/), and RSEM was used to calculate the expression of transcripts. Differentially expressed genes (DEGs) were identified by DEGseq2 with the following parameters: log_2_FC ≥ 1.00 and adjusted *P* value ≤ 0.05. Gene Ontology (GO) enrichment analysis was performed by Omicshare tools (http://www.omicshare.com/tools). Kyoto Encyclopedia of Genes and Genomes (KEGG) pathway analysis was carried out using the Majorbio cloud platform (https://cloud.majorbio.com/tool/). A summary of the sequencing data is shown in Table S3.

### Analysis of ZHD binding motifs in candidate target gene promoters

The promoter sequences of candidate target genes were entered into the JASPAR^2020^ (http://jaspar.genereg.net/) website, and the selected parameters for analysis were ZHD1 (MA1329.1, MA1329.2, AtZHD2/ATHB-22) and ZHD5 (MA1326.1, AtZHD5/ATHB-33). Detailed information is provided in Table S1. The predicted binding motif information of each target gene is detailed in Appendix S4 and Data S4.

### Dual-luciferase and yeast one-hybrid assays

Dual-luciferase and yeast one-hybrid assays were performed according to our previous study^45^. For the dual-luciferase assay, *SlZHD17*-ORF was inserted into the pGreenII 62-SK vector and transformed into GV3101 as an effector. The promoters (detailed in Appendix S4) were constructed into the pGreenII 0800-LUC vector and transformed into GV3101 as reporters. Transient expression was performed in tobacco (*Nicotiana benthamiana*) leaves, and six independent leaves were transfected. A commercial Dual-Luciferase^®^ Reporter Assay System (Promega, USA) was used to detect the regulatory relationship between SlZHD17 and these promoters.

For the yeast one-hybrid assay, *SlZHD17*-ORF was inserted into the pGADT7 vector. The promoter regions (detailed in Appendix S4) were inserted into the pAbAi vector and transformed into the Y1HGold yeast strain. The pGADT7-SlZHD17 plasmid was transformed into the recombinant yeast strain, and the empty pGADT7 plasmid was used as a control. The transformants were spread on SD/−Leu/AbA (aureobasidin A, Clontech, USA) culture medium, SD/−Leu without AbA was used as a control, and the interaction between SlZHD17 and promoter regions was determined according to colony growth. The interaction of pGADT7-p53 + pAbAi-*p53* was set as the positive control (Fig. S10).

### Electrophoretic mobility shift assay (EMSA)

*SlZHD17*-ORF was inserted into the pCold^TM^ TF (TAKARA, Japan) vector to generate a His fusion protein, purified His-SlZHD17 protein (Fig. S11) was used for EMSA, and the procedure was carried out according to the instructions of the commercial Light Shift^®^ Chemiluminescent EMSA Kit (Thermo, USA). The native and mutant probe sequences of each gene are detailed in Table S2. The position of the probe sequence on the promoter of each gene is detailed in Appendix S4.

### Bimolecular fluorescence complementation (BiFC) assay

The vectors and protocol of the BiFC assay were performed as described in our previous study^45^. In brief, *SlZHD17*-ORF was inserted into the pXY106 vector to generate an nYFP fusion protein. The ORFs of *SlMYB72*, *SlARF4*, *SlBEL11*, *SlTAGL1,* and *SlZHD17* were inserted into the pXY104 vector to generate a cYFP fusion protein. The recombinant plasmids were transformed into GV3101, which was used for transient expression in tobacco leaves. For each pair of experiments, six independent leaves were transfected and observed by confocal laser scanning microscopy (Leica, Germany) at the Analytical and Testing Center of Chongqing University.

### Firefly luciferase complementation imaging (LCI) assay

The vector information and protocol for the LCI assay were based on Chen et al.^46^. *SlZHD17*-ORF was inserted into the pCAMBIA-CLuc vector to generate a C-terminal luciferase fusion protein, and the ORFs of *SlMYB72*, *SlARF4*, *SlBEL11*, *SlTAGL1*, and *SlZHD17* were inserted into the pCAMBIA-NLuc vector to generate an N-terminal luciferase fusion protein. The recombinant plasmids were transformed into GV3101, which was used for transient expression in tobacco leaves. Three days after transfection, one millimolar luciferin (Promega, USA) was sprayed onto the injected leaves, which were kept in the dark for 6 min to quench the fluorescence before capturing the LUC image. A low-light cooled CCD imaging apparatus (Alliance, UK) was used. The corresponding 3D model of the fluorescence signal for each interaction is provided in Fig. S12. For each pair of experiments, six independent leaves were transfected and observed.

## Supplementary information

Supplementary material

Dataset 1

Dataset 2

Dataset 3

Dataset 4

## Data Availability

All relevant data are presented within the paper and its supplementary files.
