# Supplementary material for: SlZHD17 is involved in the control of chlorophyll and carotenoid metabolism in tomato fruit
Source: Hortic Res. 2021 Dec 1;8:259. doi: 10.1038/s41438-021-00696-8 (PMC8632997; doi:10.1038/s41438-021-00696-8)
Supplement: Supplementary file 1 — Supplementary material [file 41438_2021_696_MOESM1_ESM.docx]

**Table S1. The Arabidopsis ZHD binding motifs provided in JASPAR^2020^.**

| **Matrix**  **ID** | **Uniprot ID** | **JASPAR name** | **TF information** | **Species** | **Family** | **Logo** |
| --- | --- | --- | --- | --- | --- | --- |
| MA1329.2 | Q9SB61 | ZHD1 | AtZHD2/ ATHB-22 (AT4G24660) | *Arabidopsis thaliana* | ZF-HD | 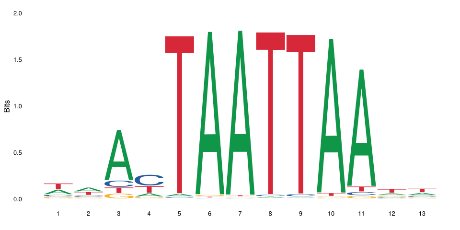 |
| MA1329.1 | None | ZHD1 | AtZHD2/ ATHB-22 (AT4G24660) | *Arabidopsis thaliana* | ZF-HD | 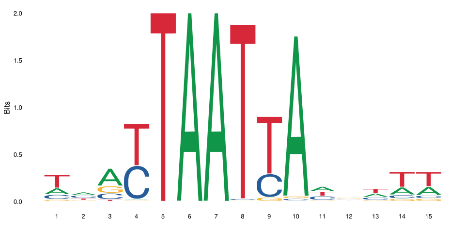 |
| MA1326.1 | Q9FRL5 | ZHD5 | AtZHD5/ ATHB-33 (AT1G75240) | *Arabidopsis thaliana* | ZF-HD | 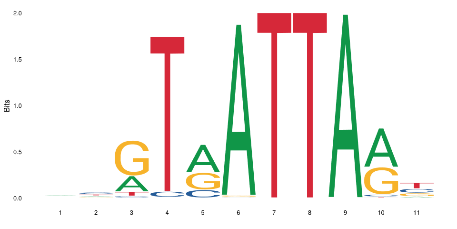 |
| <https://www.uniprot.org/uniprot/Q9SB61>  <https://www.uniprot.org/uniprot/Q9FRL5>  <http://jaspar.genereg.net/search?q=ZHD&collection=CORE&tax_group=plants&tax_id=all&type=all&class=all&family=all&version=all> | | | | | | |

**Table S2. The primers used in this study.**

| **Primers used for DNA constructs and EMSA probes** | | |
| --- | --- | --- |
| Primer Name | Forward (5’-3’) | Reverse (5’-3’) |
| SlZHD17-RNAi-sen | CGAAGCTTTGAAATGTGCTGC | TCCGCCGCTACCTCCG |
| SlZHD17-RNAi-anti | TCCGCCGCTACCTCCG | CGAAGCTTTGAAATGTGCTGC |
| SlZHD17-62SK | GGATCCAtggcattaggtggtggtaat | AAGCTTttaaggttgatcttcttgtatt |
| SlGUN4-0800 | GGTACCCTCGTTAACATTTTGATCATCC | AAGCTTGCTTAATGCTTAGTAGTATAG |
| SlCHLM-0800 | GGTACCCCTAGACGAGACAAAGGCAA | AAGCTTGTTGGAGTTGAGTTCGAGCT |
| SlPOR-B-0800 | GGTACCGTACCCTTGAATGAGGAGTGTC | AAGCTTTGGACTGATAGTGTGTGTAGT |
| SlPOR-C-0800 | GGTACCGTTGACAACTCGACGTGTCGA | AAGCTTGTCTGTAGAACTACTGGTACTG |
| SlTKN2-0800 | GGTACCCATTAAACATGACCAACTTATG | AAGCTTCTCACACACTTTCTTCTTCACTC |
| SlSGR1-0800 | GGTACCTTGACCATCCGATTAGATGCA | AAGCTTCTCCTTGAATCTTGGAGTTCC |
| SlPSY1-0800 | GGTACCGGGTAAGTTACGCATTTAGTC | AAGCTTGCAAGAAAACCTTGGTTGTC |
| SlZISO-0800 | GGTACCCATATCCAAGCAATGGAAGAG | AAGCTTTGTGAGCTCTAAGTGTGTAG |
| SlZHD17-HD-AD | GAATTCATGaagaaaagatttaggac | GGATCCgttcttattattatgcatcc |
| SlPOR-B-pAbAi | GGTACCCTTACTTTTGGTTATTGACT | GTCGACAAATAACTTAAAATAAGTAA |
| SlTKN2-pAbAi | GGTACCGTAGTATTTTTGTTAATTTA | GTCGACTTGTCCCTTAATTGAATATG |
| SlSGR1-pAbAi | GGTACCAAAAAACAAATTAGTACTTG | GTCGACTTATCTTTTTGTGCTTAATA |
| SlPSY1-pAbAi | GGTACCGAGAGTGGAAATATTCTCTA | GTCGACGCAAGAAAACCTTGGTTGTC |
| SlZISO-pAbAi | GGTACCAAAACAATAAAATTAGCTAC | GTCGACAAAAAAATAATTTTTTATGT |
| SlZHD17-His | GGATCCAtggcattaggtggtggtaat | GAATTCaggttgatcttcttgtattat |
| SlPOR-B-EMSA | AAAAATTGAAATTAGTGTATTGTGAAAAAAAA | TTTTTTTTCACAATACACTAATTTCAATTTTT |
| SlPOR-B-EMSA-mutant | AAAAATTGAAGGCCGTGTATTGTGAAAAAAAA | TTTTTTTTCACAATACACGGCCTTCAATTTTT |
| SlTKN2-EMSA | AATGTGTACGCTAATCTAATTTTTAA | TTAAAAATTAGATTAGCGTACACATT |
| SlTKN2-EMSA-mutant | AATGTGTACGCGGGGCGGGGTTTTAA | TTAAAACCCCGCCCCGCGTACACATT |
| SlSGR1-EMSA | TAAAAATATTAAGCACAAAAAGATAAAAATGT | ACATTTTTATCTTTTTGTGCTTAATATTTTTA |
| SlSGR1-EMSA-mutant | TAAAAATGGGGAGCACAAAAAGATAAAAATGT | ACATTTTTATCTTTTTGTGCTCCCCATTTTTA |
| SlPSY1-EMSA | TCTACTAGGAGTAATTTATTTTCTATAAACTAAGTAAAGTTTGGAAG | CTTCCAAACTTTACTTAGTTTATAGAAAATAAATTACTCCTAGTAGA |
| SlPSY1-EMSA-mutant | TCTACTCCCCCCCGGGGGGGGTCTATCCCCCCCCGGGGGGGTGGAAG | CTTCCACCCCCCCGGGGGGGGATAGACCCCCCCCGGGGGGGAGTAGA |
| SlZISO-EMSA | AAAAAAAAAAAATTAAATGTTTTTGAATAA | TTATTCAAAAACATTTAATTTTTTTTTTTT |
| SlZISO-EMSA-mutant | AAAAAAAAGGGCCCCGGGGTTTTTGAATAA | TTATTCAAAAACCCCGGGGCCCTTTTTTTT |
| SlMYB72-nLUC | GGTACCATGGTGAGAACAACTTGCTA | GTCGACCAAATTATAATGATCTAGATC |
| SlZHD17-cLUC | GGTACCAtggcattaggtggtggtaat | GTCGACttaaggttgatcttcttgtatt |
| SlZHD17-nLUC | GGTACCAtggcattaggtggtggtaat | GTCGACaggttgatcttcttgtattat |
| SlARF4-nLUC | GGTACCATGGAAATTGATCTGAATCATG | GTCGACAATCCTGATTACAGTTGGAGAT |
| SlBEL11-nLUC | GGTACCATGTTCCTCTTGAATTCCGTGG | GTCGACAGAAACATAATCTTGTAATAGT |
| SlTAGL1-nLUC | GGTACCATGGTTTTTCCTATTAATCAG | GTCGACGACAAGCTGGAGAGGAGTTT |
| SlMYB72-cYFP | GGATCCATGGTGAGAACAACTTGCTA | GTCGACCAAATTATAATGATCTAGATC |
| SlZHD17-nYFP | GGATCCAtggcattaggtggtggtaat | GTCGACttaaggttgatcttcttgtatt |
| SlZHD17-cYFP | GGATCCAtggcattaggtggtggtaat | GTCGACaggttgatcttcttgtattat |
| SlARF4-cYFP | GGATCCATGGAAATTGATCTGAATCATG | GTCGACAATCCTGATTACAGTTGGAGAT |
| SlBEL11-cYFP | GGATCCATGTTCCTCTTGAATTCCGTGG | GTCGACAGAAACATAATCTTGTAATAGT |
| SlTAGL1-cYFP | GGATCCATGGTTTTTCCTATTAATCAG | GTCGACGACAAGCTGGAGAGGAGTTT |
| **Primers used for qRT-PCR** | | |
| Gene Name | Forward (5’-3’) | Reverse (5’-3’) |
| *SlZHD17* (Solyc04g080490) | GCGCTGCACCACACAATAAT | CCATCCGTAACATTTCCACC |
| *SlGUN4* (Solyc06g073290) | AGTTCATTTCCGAATCTGACC | GGAATAACAAAGCGAACAGAGA |
| *SlCHLM* (Solyc03g118240) | AAGAAGGTGCCATTGTATCAG | CCATCCAAACTCTCCAAGTC |
| *SlPOR-A* (Solyc07g054210) | GGAAAAAGACTTGCACAGGTAG | CAAAAGAGGAGGAGGTACTGTT |
| *SlPOR-B* (Solyc12g013710) | TAGTAATCACTGGAGCGTCTTC | TTATCGACAAATTGTCTGACGC |
| *SlPOR-C* (Solyc10g006900) | TTCTTTCGAAAACCAGTTGTCC | GTTACAAATTCTCACTCGCGAA |
| *SlHEMA1* (Solyc04g076870) | TGTCGAGATAATCTACAAGCCC | GTTCTTTCATAAACAGTGGCGT |
| *SlALAD* (Solyc08g069030) | CAATGACAATGGATTAGTGCCC | CTTCTCTCACAATGCCATCATG |
| *SlCHLH* (Solyc04g015750) | GCTGCATACTATTCCTTTGTGG | TTTGCATAACTCCTTCGTTTGG |
| *SlCHLI* (Solyc10g008740) | CCTGCACCAAGCTGTTTTATAG | GCACAGTACACAGACAATCTTC |
| *SlCHLD* (Solyc04g015490) | GAGAGTAGCTCAAGGGAAGTAC | CCTTTGAAACCAAAATTGATGCCC |
| *SlCAO1* (Solyc11g012850) | GTTCCACAAAGTAAAGGCAAGT | CTTCCACAACCTTTTCGTGTAG |
| *SlCAO2* (Solyc06g060310) | GAATTTCGACCACCTTGTATGG | AGCAAAATCCAGTGACATCCTA |
| *SlSGR1* (Solyc08g080090) | GAGATGAAGTTGTTGCAGAGTG | CCATGAACAAAAGCCTTGAGAA |
| *SlTKN2* (Solyc02g0811200) | CCATATCCATCGGAATCTCAG | TGGTTTCCAATGCCTCTTTC |
| *SlCUL4* (Solyc02g021470) | CATCATCCTCATCTACTCCGAC | ATATCGGAGGAAAAGTGAACGT |
| *SlDDB1* (Solyc02g021650) | GCCCATAACACTTCGTACATTC | GTGCCAATTGTTAACTCACCTT |
| *SlBL4* (Solyc08g065420) | GGATATAAGTTGCTCCGGTGTA | TGTGAAAGGTAGACAGGTTTGT |
| *SlTKN4* (Solyc01g100510) | ACATAGAGTCACAGCTCAGTTC | CATTATCGCCTTCACAGTTAGC |
| *SlGLK1* (Solyc07g053630) | CCGTAAGCAGTGGTGATGAGTCTG | AACCCGAACCTACATCCGAAGC |
| *SlGLK2* (Solyc10g008160) | CCTTACATGTTTGGGGGCATCCAC | GGGGTGCAAATCAGAGGC |
| *SlAPRR2-like* (Solyc08g077230) | GGTGACTGCCATGATCATACTA | GTTTCGACGGATTTAGTGTCAG |
| *SlNAC4* (Solyc11g017470) | CACGTTTACAATGACTTCACGT | TATCTTGTAGCGGTGACATCTC |
| *SlPSY1* (Solyc03g031860) | GGAAAGCAAACTAATAATGGACGG | CCACATCATAGACCATCTGTTCC |
| *SlPDS* (Solyc03g123760) | GGTCACAAACCGATACTGCT | AAACCAGTCTCGTACCAATCTC |
| *SlZDS* (Solyc01g097810) | AGTGGTTTCTGTCTAAAGGTGG | ACCGAGCACTCATGTTATCAC |
| *SlZISO* (Solyc12g098710) | CCTTCTTCTTCCTATACCCGTC | TGCCTGGTAATCCTCATAATCC |
| *SlCRTISO* (Solyc10g081650) | TTTTGGCGGAATCAACTACC | GAAAGCTTCACTCCCACAGC |
| *SlCYC-B* (Solyc06g074240) | TGTTATTGAGGAAGAGAAATGTGTGAT | TCCCACCAATAGCCATAACATTTT |
| *SlZHD2* (Solyc01g102980) | AAAACAAGAAGAAGGGGTTGTG | CTTGGCAAGGCTATGCTTATTG |
| *SlZHD10* (Solyc02g085160) | GGTGTGCATAGAAATAACCACC | GAAACTCATCATTACTGGTGCC |
| *SlZHD22* (Solyc09g089550) | TGTCCCAGAAGTAACTCATTCC | ACTGCAACTAATTCCTACGGAA |
| *SlActin* (Solyc11g005330) | TGTCCCTATTTACGAGGGTTATGC | CAGTTAAATCACGACCAGCAAGAT |
| The qRT-PCR primers of *SlGUN4* was referenced to Bassa et al. 2012; *SlCHLM* and *SlTKN2* were referenced to Meng et al. 2018; *SlGLK1* and *SlGLK2* were referenced to Powell et al. 2012; *SlPSY1*, *SlPDS*, *SlZDS*, *SlCYC-B* were referenced to Liu et al. 2018; *SlCRTISO* was referenced to Fraser et al. 2007.  Bassa et al. Phenotypes associated with down-regulation of *Sl-IAA27* support functional diversity among Aux/IAA family members in tomato. 2012, Plant Cell Physiology, 53(9): 1583-1595.  Meng et al. BEL1-LIKE HOMEODOMAIN 11 regulates chloroplast development and chlorophyll synthesis in tomato fruit. 2018, The Plant Journal, 94, 1126-1140.  Powell et al. *Uniform ripening* encodes a *Golden 2-like* transcription factor regulating tomato fruit chloroplast development. 2012, Science, 336, 1711-1715.  Liu et al. The tomato ethylene response factor Sl-ERF.B3 integrates ethylene and auxin signaling via direct regulation of *Sl-Aux/IAA27*. 2018, New Phytologist, 219 (2): 631-640.  Fraser et al. Manipulation of phytoene levels in tomato fruit: effects on isoprenoids, plastids, and intermediary metabolism. 2007, The Plant Cell, 19: 3194-3211. | | |

**Table S3. The overview of RNA-seq data quality.**

| **Sample** | **Raw reads** | **Clean reads** | **Error rate (%)** | **Q20**  **(%)** | **Q30**  **(%)** | **Total mapped** | **Uniquely mapped** |
| --- | --- | --- | --- | --- | --- | --- | --- |
| WT_MG_1 | 48276188 | 47868712 | 0.0244 | 98.28 | 94.73 | 46119295(96.35%) | 45364866(94.77%) |
| WT_MG_2 | 51133286 | 50683242 | 0.0245 | 98.24 | 94.62 | 49045727(96.77%) | 48256025(95.21%) |
| WT_MG_3 | 52358654 | 51902194 | 0.0246 | 98.21 | 94.54 | 50159983(96.64%) | 49293731(94.97%) |
| RNAi_MG_1 | 52753932 | 52319264 | 0.0245 | 98.23 | 94.62 | 50699528(96.9%) | 49909948(95.39%) |
| RNAi_MG_2 | 59686528 | 59146102 | 0.0246 | 98.21 | 94.55 | 57291536(96.86%) | 56414754(95.38%) |
| RNAi_MG_3 | 52082910 | 51635774 | 0.0244 | 98.28 | 94.73 | 50066681(96.96%) | 49285090(95.45%) |

**Figure S1. Amino acid sequence alignment of SlZHD17 and JASPAR^2020^ AtZHDs.**

The amino acid sequences of SlZHD17, AtZHD2 (ATHB-22), AtZHD3 (ATHB-21) and AtZHD5 (ATHB-33) are detailed in the following Appendix S2, S3.


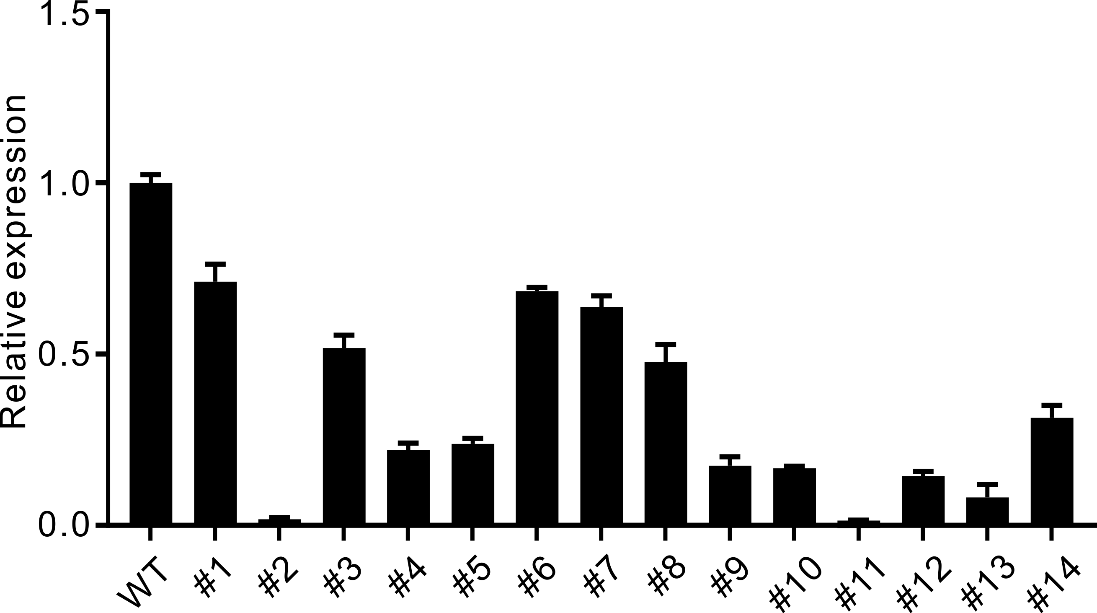


**Figure S2. Identification of *SlZHD17*-RNAi plants.**

The relative expression level of *SlZHD17* gene in WT and T0 generation RNAi leaves. Data represent the mean values of three independent experiments and error bars show the ± standard error values.


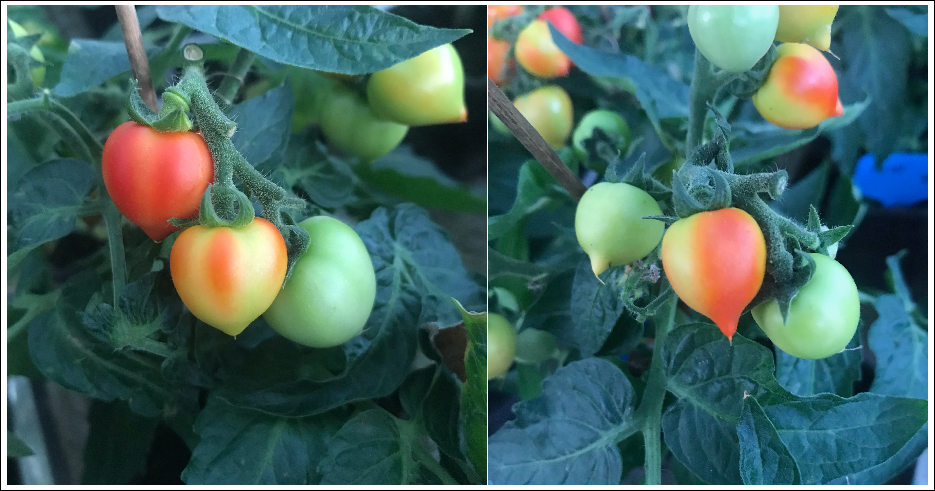


**Figure S3. Photograph of *SlZHD17*-RNAi fruit on the plant.**


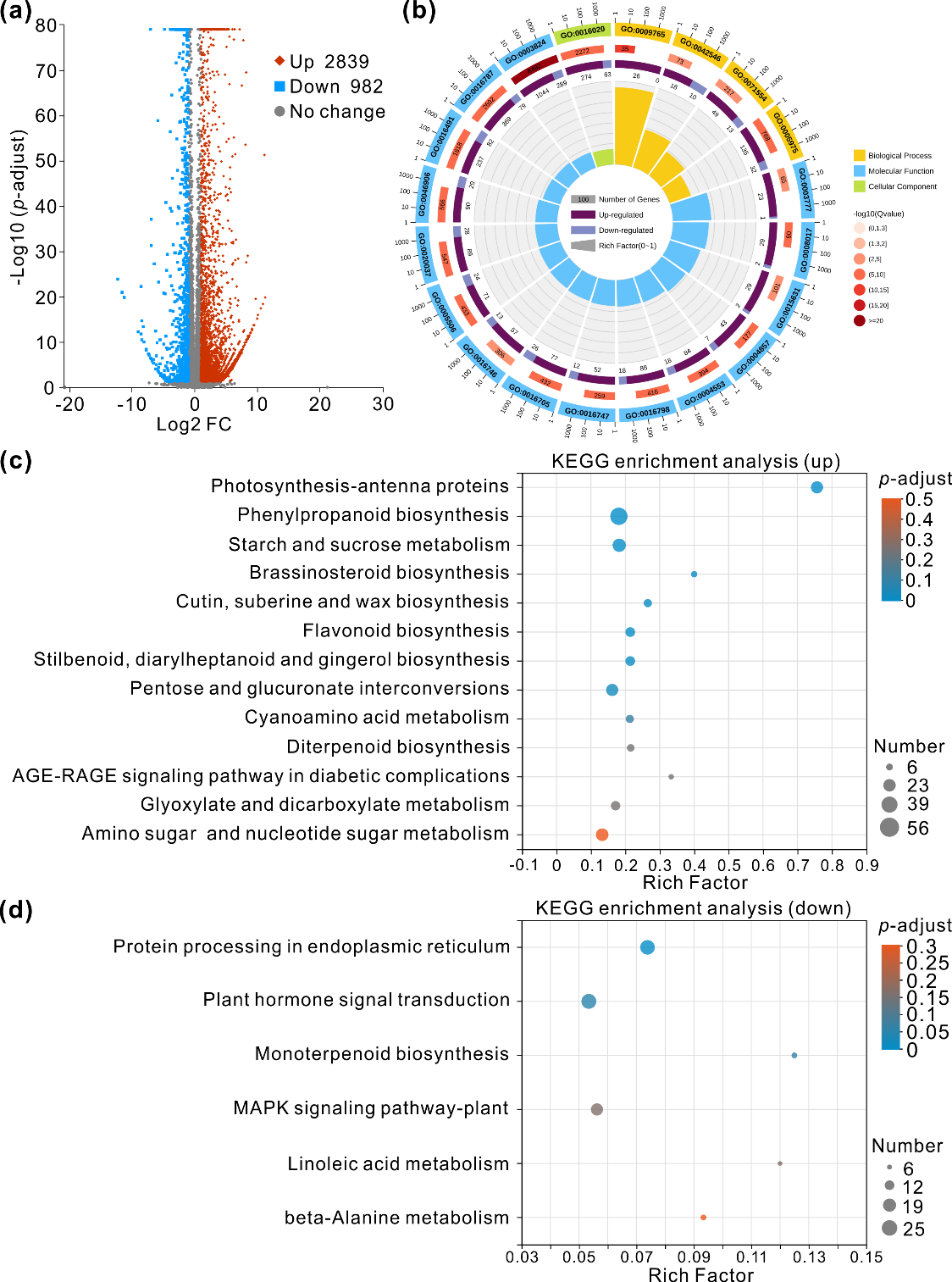


**Figure S4. RNA-seq analysis of WT and *SlZHD17*-RNAi fruit at the mature green stage.**

**(a)** The number of differentially expressed genes (DEGs) between WT and *SlZHD17*-RNAi fruit at mature green stage. log_2_FC ≥ 1.00 and adjusted *P*-value ≤ 0.05.

**(b)** Top 20 most significantly enriched gene ontology (GO) terms for DEGs.

**(c, d)** The most significantly enriched kyoto encyclopedia of genes and genomes (KEGG) pathways for up-regulated genes **(c)** and down-regulated genes **(d)**.


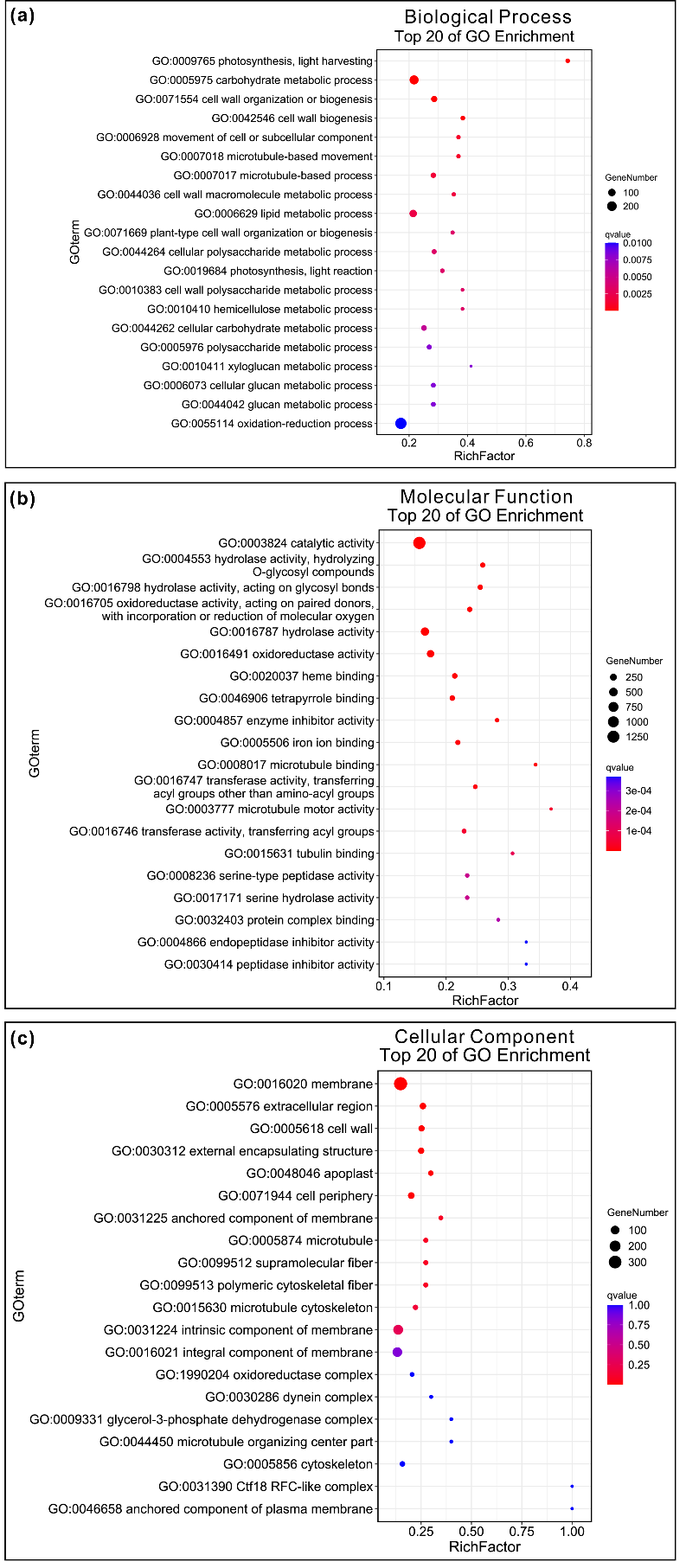


**Figure S5. GO enrichment of DEGs between WT and *SlZHD17*-RNAi fruit at the mature green stage.**

Top 20 GO enrichment terms of biological process **(a)**, molecular function **(b)**, and cellular component **(c)**.


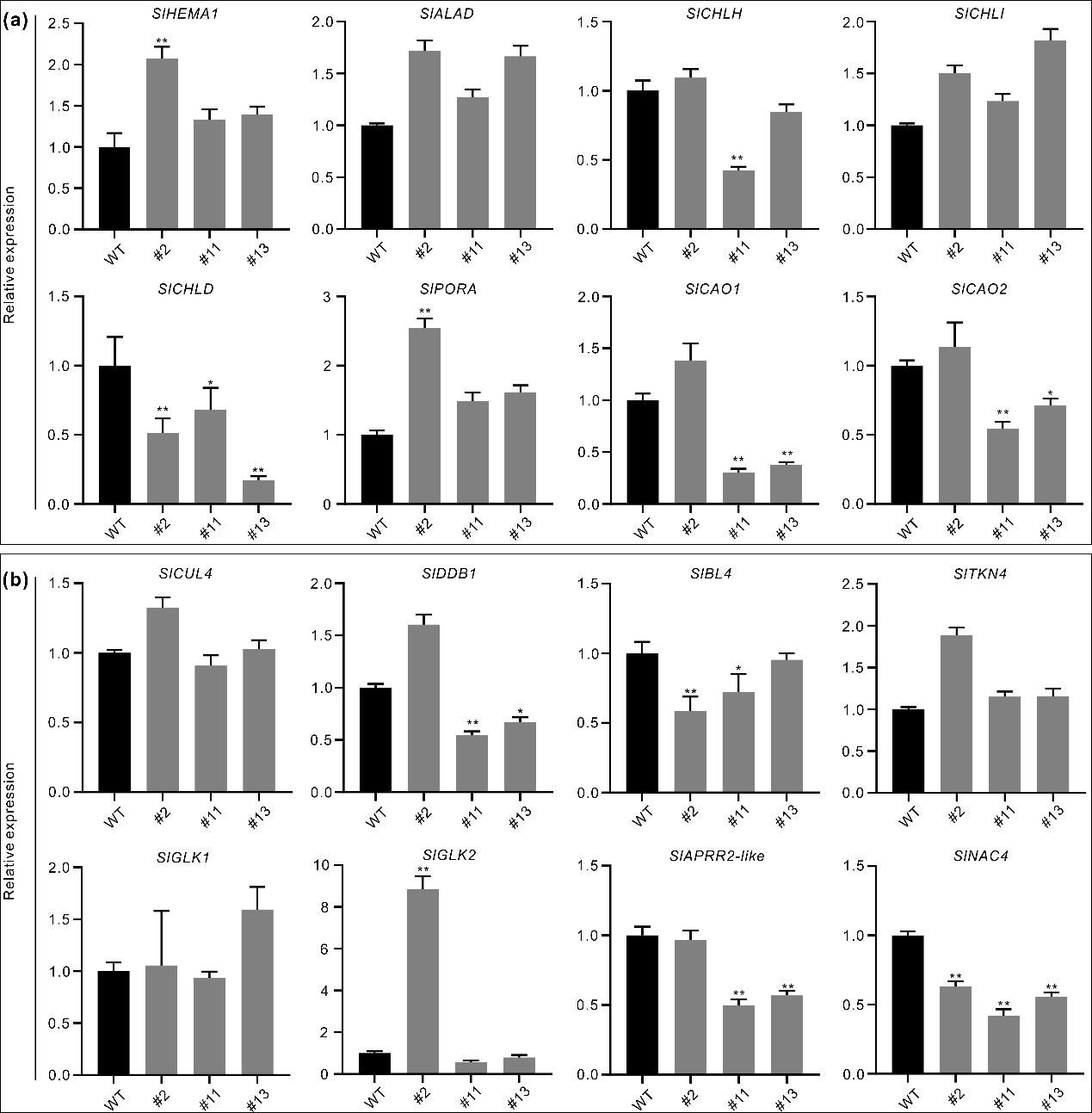


**Figure S6. The relative expression levels of chlorophyll biosynthesis and chloroplast development genes in WT and *SlZHD17*-RNAi fruit at the mature green stage.**

The relative expression levels of chlorophylls biosynthesis genes **(a)** and chloroplasts development genes **(b)** in WT and *SlZHD17*-RNAi fruit at mature green stage. The transcript level in WT was set as 1, data represent the mean values of three independent experiments and error bars show the ± standard error values, ** refers to significant differences between WT and transgenic lines with *P* < 0.01 (two-tailed Student's *t*-test).


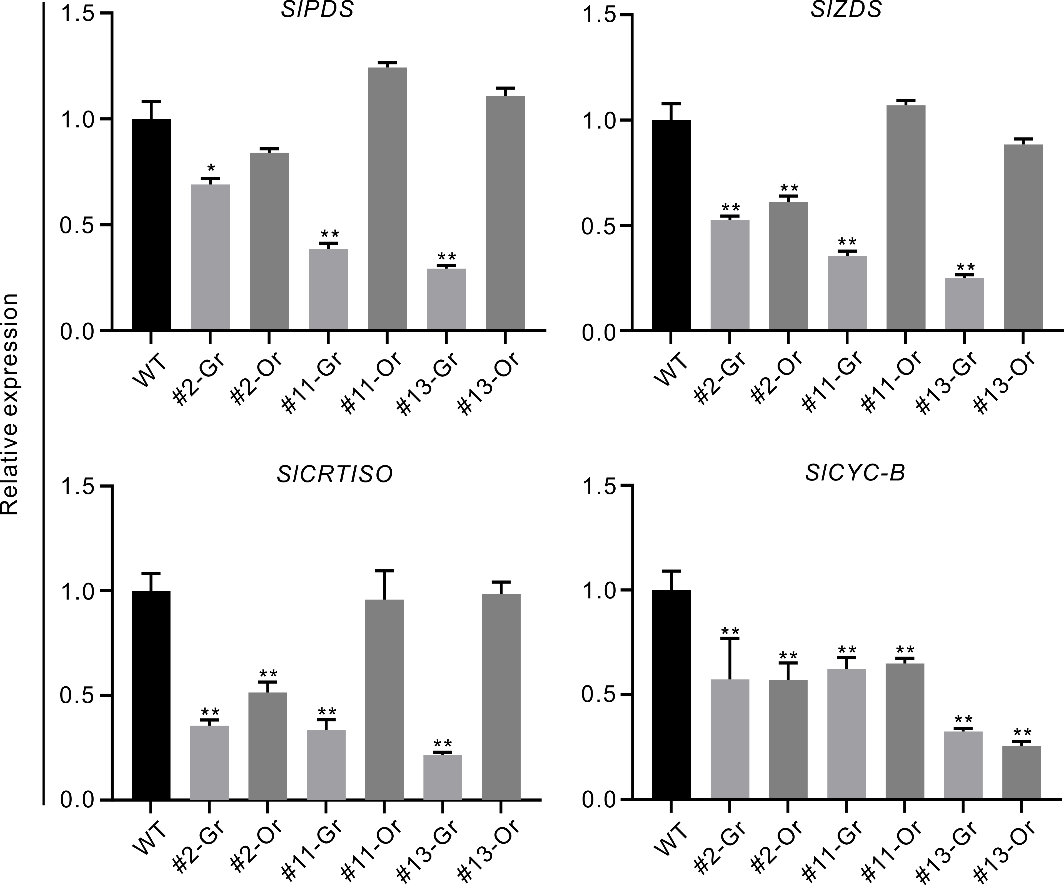


**Figure S7. The relative expression levels of carotenoid biosynthesis genes in WT and *SlZHD17*-RNAi fruit at the Br+3 stage.**

The relative expression levels of carotenoids biosynthesis genes in WT and *SlZHD17*-RNAi fruit at Br+3 stage. The uneven pigmentation RNAi fruit were divided into normal orange (Or) region and unnormal green (Gr) region. The transcript level in WT was set as 1, data represent the mean values of three independent experiments and error bars show the ± standard error values, * and ** refer to significant differences between WT and transgenic lines with *P* < 0.05 and *P* < 0.01, respectively (two-tailed Student's *t*-test).


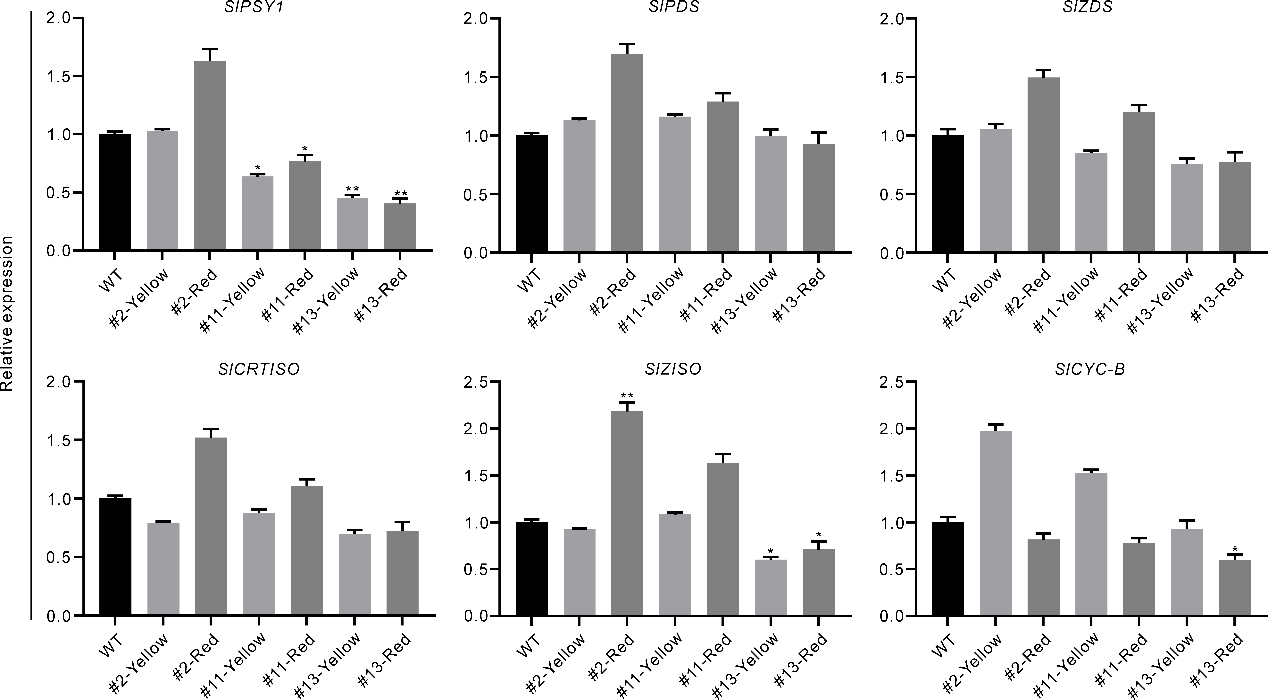


**Figure S8. The relative expression levels of carotenoid biosynthesis genes in WT and *SlZHD17*-RNAi fruit at the Br+7 stage.**

The relative expression levels of carotenoids biosynthesis genes in WT and *SlZHD17*-RNAi fruit at Br+7 stage. The uneven pigmentation RNAi fruit were divided into normal red region and unnormal yellow region. The transcript level in WT was set as 1, data represent the mean values of three independent experiments and error bars show the ± standard error values, * and ** refer to significant differences between WT and transgenic lines with *P* < 0.05 and *P* < 0.01, respectively (two-tailed Student's *t*-test).


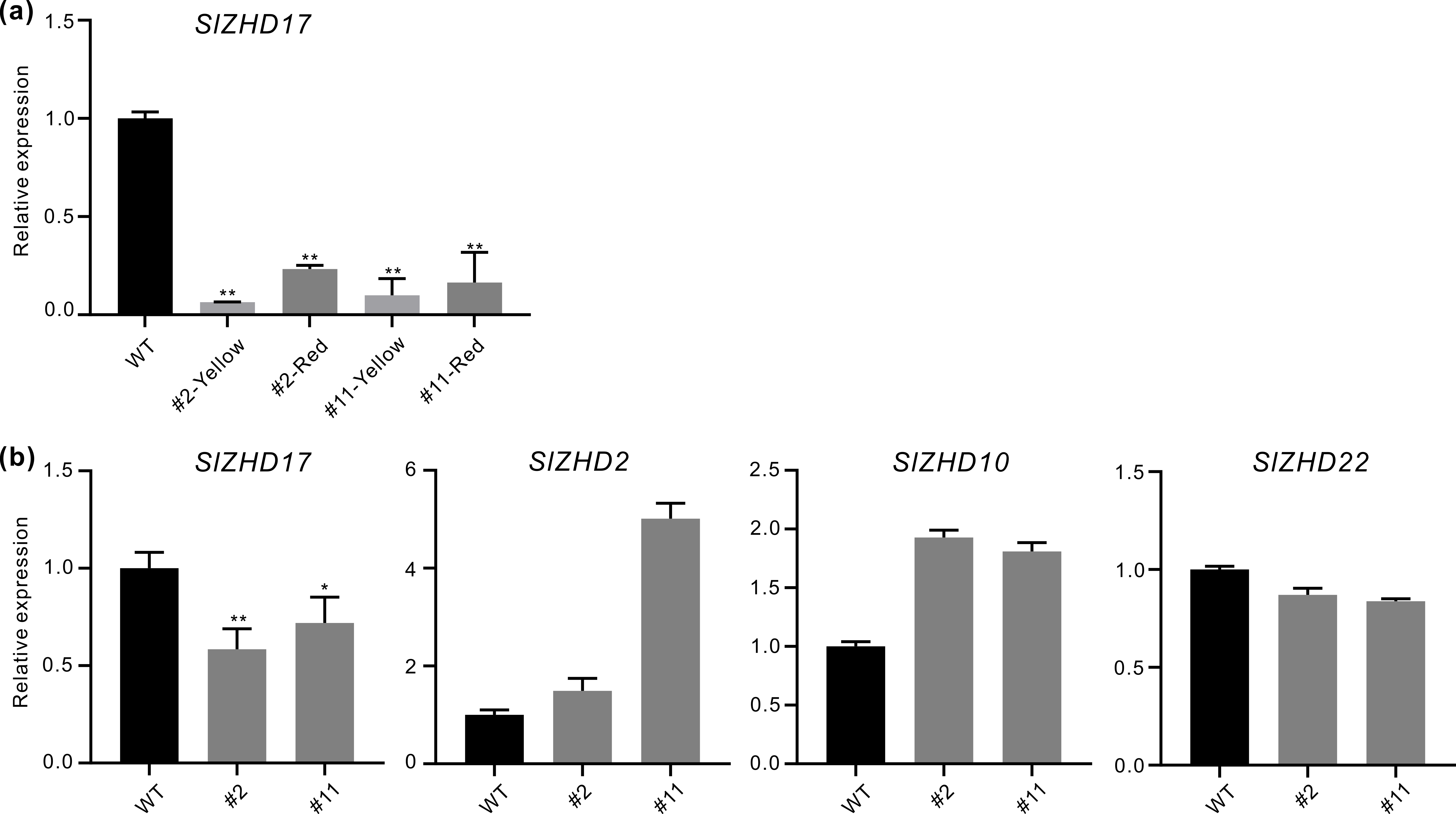


**Figure S9. The relative expression level of *SlZHD17* and its homologous genes in WT and *SlZHD17*-RNAi fruit.**

**(a)** The relative expression level of *SlZHD17* gene in WT and *SlZHD17*-RNAi fruit at Br+7 stage. The uneven pigmentation RNAi fruit were divided into normal red region and unnormal yellow region.

**(b)** The relative expression level of *SlZHD17* and its homologous genes in WT and *SlZHD17*-RNAi fruit at mature green stage.

The transcript level in WT was set as 1, data represent the mean values of three independent experiments and error bars show the ± standard error values, * and ** refer to significant differences between WT and transgenic lines with *P* < 0.05 and *P* < 0.01, respectively (two-tailed Student's *t*-test).


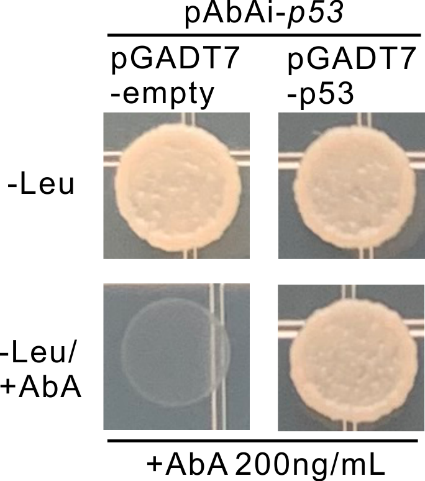


**Figure S10. The positive control of the yeast-one hybrid assay.**

The interaction of pGADT7-p53+pAbAi-*p53* was the positive control. SD/-Leu without AbA (Aureobasidin A) as control.


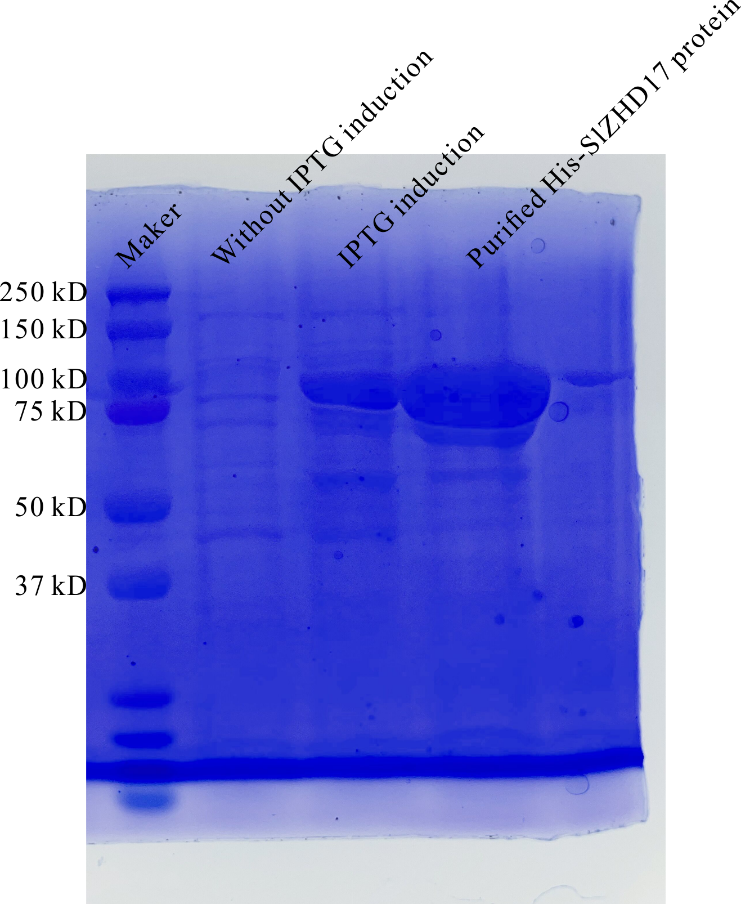


**Figure S11. Purified His-SlZHD17 protein was used for the electrophoretic mobility shift assay.**

The recombinant pCold^TM^ TF-SlZHD17 vector fusion expressed a Trigger Factor (TF) tag that promotes the induction of soluble protein. The full length SlZHD17 protein was 31.06 kDa, and TF tag was 48 kDa.


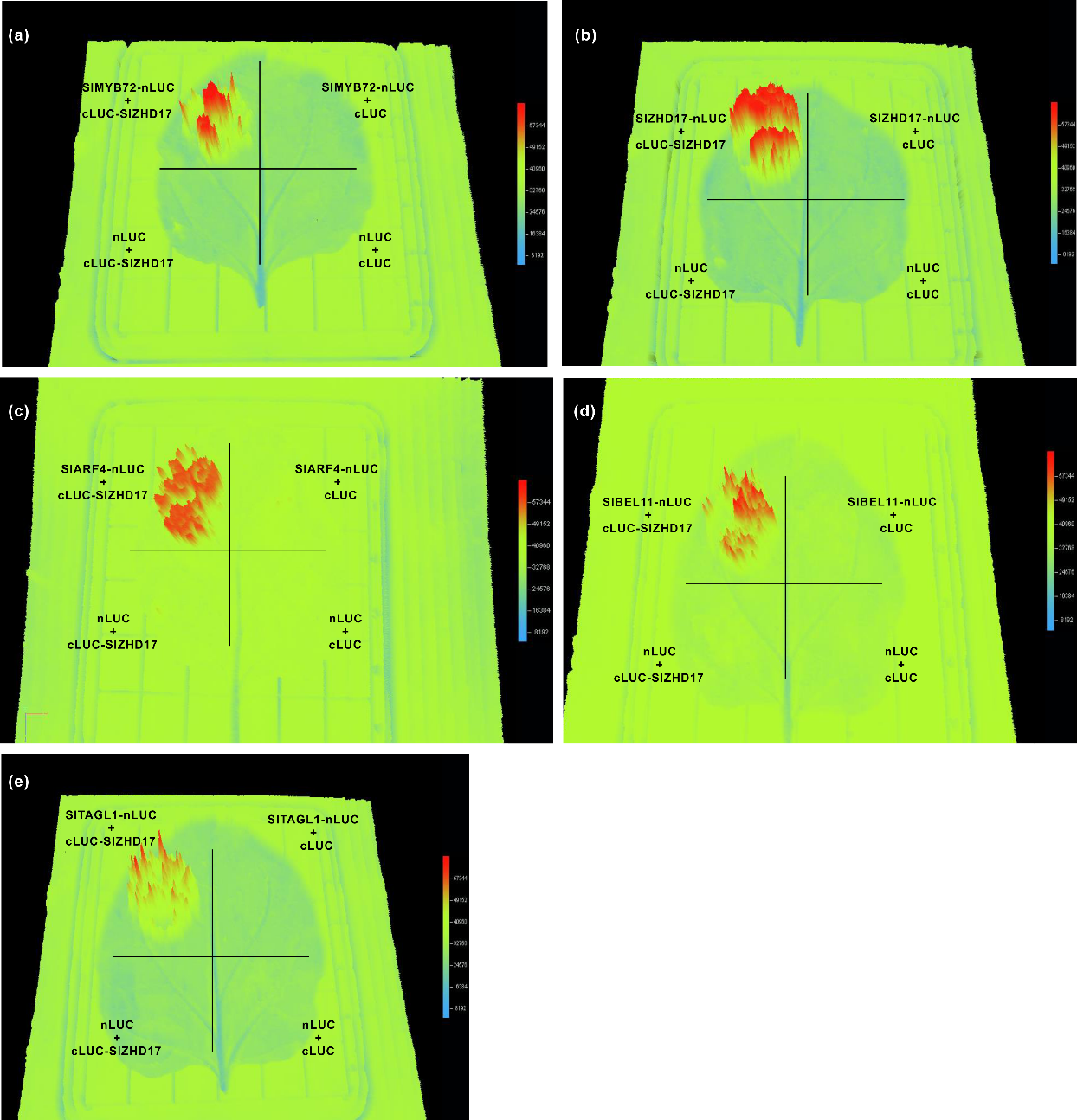


**Figure S12. The 3D model of the fluorescence signal for each protein protein interaction.**

**(a)** The 3D model of the fluorescence signal of the interaction between SlMYB72 with SlZHD17.

**(b)** The 3D model of the fluorescence signal of the interaction between SlZHD17 with SlZHD17.

**(c)** The 3D model of the fluorescence signal of the interaction between SlZHD17 with SlARF4.

**(d)** The 3D model of the fluorescence signal of the interaction between SlZHD17 with SlBEL11.

**(e)** The 3D model of the fluorescence signal of the interaction between SlZHD17 with SlTAGL1.

**Appendix S1. The open reading frame sequence of the *SlZHD17* gene.**

The sequence with single underline is used for RNA interference (RNAi) vector construction.

atggcattaggtggtggtaatgaggaaaaagagatgagaatttcaggttttcaaaatatagatcttgtctcacctggtggtggtgcagcgccagacgacatcgtcggcgctgcaccacacaataattccactactggatctaataaattgaacaagggaactagcacagtgagaggtagatatcgtgagtgcttgaaaaatcatgctgctaatattggtggaaatgttacggatggatgtggtgagtttatgcccagtggtgaagaaggtacactcgaagctttgaaatgtgctgcttgtaattgtcataggaattttcatagaaaagaaatacctaacaacgttggcgttggtggtgataataataatgcggggattatggttgtgcatccattacagctaccacaaccactaccttctccgataccgtcgttgaatcatcatcaccaccatcaccatcaacacggaggaagatcaatttggactactatgccacctcagcctgtcaaaatggccttcggaggtagcggcggaggaagtggggcaacagactcatcaagtgaagaacttaatttcaacacatatcatcatcaacaagcaacatcagtacctccacaacaaccatttatgttggctaagaaaagatttaggacaaaatttagtcaagaacaaaaggaaaaaatgttggaatttgctgaaaaattaggttggagaattccaagagaagatgatgctgaagtacaaagattttgctcacaagttggagtcaagagacaggtttttaaggtttggatgcataataataagaacccttcttctgctaaaaaaaatataatacaagaagatcaaccttaa

**Appendix S2. The amino acid sequence of SlZHD17.**

The amino acid sequence with single underline is zinc finger domain (60-112aa), the amino acid sequence with double underline is homeodomain (213-269aa).

MALGGGNEEKEMRISGFQNIDLVSPGGGAAPDDIVGAAPHNNSTTGSNKLNKGTSTVRGRYRECLKNHAANIGGNVTDGCGEFMPSGEEGTLEALKCAACNCHRNFHRKEIPNNVGVGGDNNNAGIMVVHPLQLPQPLPSPIPSLNHHHHHHHQHGGRSIWTTMPPQPVKMAFGGSGGGSGATDSSSEELNFNTYHHQQATSVPPQQPFMLAKKRFRTKFSQEQKEKMLEFAEKLGWRIPREDDAEVQRFCSQVGVKRQVFKVWMHNNKNPSSAKKNIIQEDQP*

**Appendix S3. The amino acid sequences of AtZHD2, AtZHD3 and AtZHD5.**

AtZHD2/ATHB-22 (AT4G24660):

MNFEDQEEDMEMSGVNPPCGYDSLSGEGATSSGGGGVGRSKGVGAKIRYRECLKNHAVNIGGHAVDGCCEFMPSGEDGTLDALKCAACGCHRNFHRKETESIGGRAHRVPTYYNRPPQPHQPPGYLHLTSPAAPYRPPAASGDEEDTSNPSSSGGTTKRFRTKFTAEQKEKMLAFAERLGWRIQKHDDVAVEQFCAETGVRRQVLKIWMHNNKNSLGKKP

AtZHD3/ATHB-21 (AT2G18550):

MNNQNVDDHNLLLISQLYPNVYTPLVPQQGGEAKPTRRRKRKSKSVVVAEEGENEGNGWFRKRKLSDEQVRMLEISFEDDHKLESERKDRLASELGLDPRQVAVWFQNRRARWKNKRVEDEYTKLKNAYETTVVEKCRLDSEVIHLKEQLYEAEREIQRLAKRVEGTLSNSPISSSVTIEANHTTPFFGDYDIGFDGEADENLLYSPDYIDGLDWMSQFM

AtZHD5/ATHB-33 (AT1G75240):

MDMRSHEMIERRREDNGNNNGGVVISNIISTNIDDNCNGNNNNTRVSCNSQTLDHHQSKSPSSFSISAAAKPTVRYRECLKNHAASVGGSVHDGCGEFMPSGEEGTIEALRCAACDCHRNFHRKEMDGVGSSDLISHHRHHHYHHNQYGGGGGRRPPPPNMMLNPLMLPPPPNYQPIHHHKYGMSPPGGGGMVTPMSVAYGGGGGGAESSSEDLNLYGQSSGEGAGAAAGQMAFSMSSSKKRFRTKFTTDQKERMMDFAEKLGWRMNKQDEEELKRFCGEIGVKRQVFKVWMHNNKNNAKKPPTPTTTL

**Appendix S4. The promoter sequences of candidate target genes.**

The sequence highlighted in green is the ZHD recognition site analyzed in JASPAR^2020^. The detailed position and sequence of the predicted ZHD recognition sites are also provided in Data S4.

The sequence with single underline is used for yeast-one hybrid assay, the sequence in red font is used for EMSA, the double underlines indicate the primers used for the pGreenII 0800-LUC vector construction.

***SlPOR-B* (Solyc12g013710):**

GTACCCTTGAATGAGGAGTGTCAAGAGACACCCTCCACTAGAAAGTTAATTATCTTACTAAAAAGTTTCATTTTATTAAATTAAGAAAAAATATTAATTAAATATTTTTTGTTTTATCCTTACATTTATTTATAAAGTGACTTAAAAACATTAAATAAAAATTGTTGAAATATTAACTTAAATTCTTTTGAATAGCAAGTACTACTCCATGTGACAATGGAGTTTAATAACTGCCCTTTTATCCCCTGTCCCTGTCACTAGTAGATTTCCCATGATGAAGCCCATGACTAAATATAAGAGGATTGCCTTGGGTAAAGAGCCAAATATCTCCATTTTTTTTTGAATTAAAAGAGTGTTTCTGAATTATTTTTAAAAAAAGAATTTCGCCGTTTAAGAAGAATAATTTAGTCTGACTTAATAAGTAATCAAAGATAGTAAAAAAGTTTTTTTTTATCTTTGTATCATAAAATAAAGTTGTATCAAATGTTTTAGAATATACTTTAATTTCGTCACCTTAAATATGTCGCATGAAAAATTGAAATTAGTGTATTGTGAAAAAAAATGAGTCATTTTTTAAAAAATAGACTCAGGGCCCGTTTAAATTGACTTTGAAAAAGTAGCTTTTAAATCAAAAAGAAAAATTCAAACTGAAATGACTTTTAAATCAAAAGAATAAAAGGTAGGGGAAACTTACTTTTGGTTATTGACTTATTTTAAGTCATTCTTGACCTTGCCAAATATTTGTTAATTTATTTTAAATCATTTTTTTACTTATTTTAAGTTATTTTTATATTTGCAAACACTTACATAAATCGAAAACCGACGTAAAAGTAAATTTTATCAACTTTTAAACCAATTCAAACACCCTCTAAAAGAAAAGCAGGTCAATATTTTTGAAATGAAAAGAGTTATATACATTTTCAATTCAATAAATCATATTTAAAAATTAAAGTATGTTTTGCCATGAATATAAACTAAGTTGTTTTCGGAAATTCAAATTCTAAAATTTTATGGCCAAATATGTTTTCGTGAAGTTCAACTCTAGAAAAAAAAATGATAAATTTTCCTAAGCAAACGGACCCTAAATAACATTATGACATAGAAATATGTTTGTTATTTTAACTTTATTTATCTGATTTTAATTTAACAAAAGATTTTTATTTTTTAAAAAAATCGAAAAATTGAGCATTACAATATATTTTATTGTAGAAATATCAAAAGGAAATCTAAACATTGAAGCCTGATTGGTGGCTTACAATTGACACAACTCATAAATATTTATCTTATCCAAAATGATACTCTATTTTGTTTTTGTCTAAATATCATGTAACACCCCATAATATCTCAACTATAAATATAATTGTCCCAAATTCTTACTACACACACTATCAGTCCAAAAATCATTTTTTAAAATCTCCATCTTACAATTTTTTCAAAACAAAAATCATTTTCCTAATATAATC**ATG…**

***SlTKN2* (Solyc02g081120):**

CATTAAACATGACCAACTTATGATTTTGTCTGATTTTAATGTTTTTTAGGTGATTAGCAATCTAAGTAATTTATTTTGAAAGTGCTTATAAAATTAAGATGGAAATTTGAAAATATCAGTTTTTGAAGTTTGAATGGTGTGTGAAATATTTTTGTAGGATTTAAAACAATACATAGGATAAGTGAAATGAAATTTCATCCAAATTTATGTAAATTGTCAAAAATTGATTAAAGTTTATGAACAAAAAACAATTTGTAATTTCTTTAAAAATTCTTCCTCCAAAATCTAGAGAAAATGTCATTGACGGTTTCTTAACTATTCGAGTAGGGTTTAAATAGTTTCTTAACTATTCATTTATAGGATTTAATCTTTCTAATTGCTCAAAATATTAATATATTTGATCTTTTAACTATTTACTTACCGATCGATTTTGGTCAGACCTACCATAGAGCTTAGTCTCTTAAAGATGTGAATCTCAAGATTTTCTACTGCTATATCCACTCACATCACAATTCACACCTTTGTTGTGCGTCTATTTTTTTCCCCTCTCCCTCTAAAAAAGATAAGTTTTCTTCACGTTTTATTCTTGAAAAGTAGTATTTTTGTTAATTTACTACTAGTATAATATAATGTGTACGCTAATCTAATTTTTAATATAAAATAATGATATAAAGTTTAATTTAGTGGTTGAAAGAAGAAGCAAAGCAAAAAGAATCATATTCAATTAAGGGACAAATAGGTCTCAAGTTATAATATTGGTAAGGAACTCTCACTCTTGTCTGTTTTCCAATAACCCAAAAACCAAGAAACACATAAAAACACAGAAGAAATTACACTGCACCTCGATTTGATCATTCTAATCTAAAAATAAAAATTAAACTGTATATCTCTCTCTCACTAGGAGAGGAGGGATATTGGCAGATAATGACAGTGTTTCAGTGGCAGTGAAGAAAAAAGGGGTATGAGTTTTCATTGGGAAGGTAAAAATTTTGCACCCAAATACAAGCTAACCCTTTAACATGTTCATGTTTTTCATAGTCTTTGCTTCTTGCTACAACACTATAGAGAGAAAAAAAAAAGAAAAGAAAAGTGAACAATACACTGTTTTTTACTAATTATTTTTTAGAAAAAGAAAAAAGGAATATTGTGTGTTTGCTTTTTTTTCTGACTAGTAGTATTGCTAACTATGTATTCCATTAAGGATTTGCTGTGAAAAAGCCTGATATCAGTAAGCATAAAACTCGGGAGATCACTTACACACACACACCCTCGTAAAAAAGAGAAGAGAGATTTACTGTTAAACAGAGGTTTTTTTCCATTTCTTTTTTTTTTCTCAGTGTGTGTGAGAGAGAGAGATGGTTTTCATAGGCAAAAACAAATAGAAAGGAACAAAATTTAGAGTGAAGAAGAAAGTGTGTGAGAGAATA**ATG…**

***SlSGR1* (Solyc08g080090):**

TTGACCATCCGATTAGATGCAAATCTTGTACTAACTCACACATCACAACATGGAAGGTGGTGTAAGCAAACTTCACTAACGCCTCACATTACAATTTCTTCTAATTTCAATACCTATACGCAATTCCTACTTTTGTCTCTTTCTAGCCTTTTCCACATCTCTCTAATGACTTTTATTTTCTTTCACACACAGTGACACAAATTATTAGTACACGTACTCGGATAAAATTGTTTCTTTCTTAATTAGGGTATCAGATTTTAGTTTAACGTATGAAAAAATTCTTGTTAAAAAGGGTTTTTTCAAATAGCATATTGGTAGATTCAGATCGATCGAGCTCCAATACGAATATCGGAATAAGAAAAAAAAAAAAACAAATTAGTACTTGTAATGAATGGCCAATCTTCAAAGTCACTAAAGCCTAAAGCTTAAAGCAAAAGAAACTAGGGCCCGTTTGGATAGGCTTAATAAAAGCAGCTTTAAAAAAATACTTTTAAAAGTGCTGAAATTTATTTTTAAAATAAGCAGTTATGCGTGTGCTGAAGTTGTTATTTCAAACGTGAAAAGGAAAAAATGGGAGAAAGAAATGTTAGGGGTTATATGGGTAATTTGGAGATTGTATAAAAATATTAAGCACAAAAAGATAAAAATGTGGTCAACTTAAAACAGCTTATAAGCTAAAAAAGAAAAGCCCCTACCCCAGCCTTTAACTTTTGGCTTAAAATAAGTTTTTTTAAACTTAAAATAAGTTGTTTTGAGTATTGCCAAACAGCTAAATAAGTCAAAAACCAGCTTTTAAGTCAGTTTGACCAGCTTTTAAGCTGAGCCAAACAGGCTTAGGGCCCGTTTGGATGGGCTTAATAAAAGCAGCTTTAAAAAAGTACTTTTGAAAGTGCTGAAACTTATTTTTAAAATAAGCAGTTATGCGTTTGGATAAAAATGCTGAAGTTGTTATGTCAAACGTGAAAAGGGAAAAAATAGAAGAAAGAGATGTTAGGGGTTATATGGTTCATTTGGAGATTGTATAAAAGTATTAAGGGAAAAAACATAAAAATATGGTCAACTTAAAACAACTTATAAGCTAAAAAAAAAACACATCTACCCCAACTTTTAACTTTTTGCTTAAAATAATTTTTTTTAAACTTAAAATAAGCTATTTTGAGTATTGCCAAACAGTTAAATAAGTCAAAAATCAGCTTTTAAGTCAGTTTGACCAGCTTTTAAGCTGAGCCAAACGGGCTCTTAAATAAACTTCTTCCCAACTCTCTGTGAGGACTTTTATCAAACAGCTAACTTGCAATTTCTTTTATATACTTTTAACATTCAACAAGATTGTTTTATTACTGGAAATTTCCAGTAATATTGGAACTCCAAGATTCAAGGAGTTTTGGGTACCCAATTTCTTGTAGAAAAA**ATG…**

***SlPSY1* (Solyc03g031860):**

GGGTAAGTTACGCATTTAGTCGTAACTTTAGTCAAACTTCGTAATAATTTAGTAAGTTAAAATATATTAGAAATTTTCAGAATTCATAAACTTTAAATTTTAAATTTTGACTTCGCTTTGTGTGACTATACAATTACAGAAATTCAGAGTGGCCATTGTTGAAAGAGAGGGTGGAATTTGTGTAAGTTTTGTTTCCTTTCAGTTCTTGATATATAAAGTTGCAATCTTTAACATTCTTTGTTCACTTTCTATAGGTTTGCTAGGTTCGGTTAAATTCAGTAGCTTTAGTTTAAACCCTATGCGGAATAGAGAATGTGTAAACTTTAAACTTCAAATTTTGGCTCCGCATACGACTAGCGACTATATAATAATAGGAATTGAGCACTTGGCTTTTGTATATAGCTTCTATGTGTACCAAAATTAGAAAATCAGGCGATTATTATAATCTTGTTGACTAAATATAGAATGCATCCATTACCCCCAAAAAGTGTGATTCCACTGTCATAGGAGGTTTTTTTTATTTCATTTTATTTGTGCTTTCAATAATGTAGAGTAGTTTTACAAAGATCCTTTCTTTGTGACACATGGTAGGTAATATTGCTGATTTTGTTGTAGTTTTGGGGTTATAAAGTTTCAAATTATTTATACTGGAGGGTAGGGGTGGGGGTTGTCTATAATGCAGGTTATGGTTTTACGTGAACTCAATAATTATTGTAGATACTAAGAAATCCACTCAGTGTTCTTGCGGTGTCTTGCTTTTGATTTCAGCATCACTTGTAGTTGATTGTGTTTAGATTATCACATTATTCTGTGGCTGTAACTGTATCCTTGTTAGTTGCTTTGTTTCTACACTGTTGTTTTCCCTCTTTTATACCTATTTTGATATGTTGTACTCGAACGAGGGTCATCGGGGAACAACCTCTTTACCTCCGTGAGGTAGAGCTATGGTCTGTGTCCACTCTACCCTCCCCAGATCCCTCTTGTAGGATTTCACTATATTGTAATATTAACTTGAGGTCACTATAGGAGCTCAAAAACTTCTAATTTTGAATCAATGTCTGGTTATACTTTTTTTGTCATAACTGTATCTCAAATGTGGTGTTTGGTTTATCTCATTTTGCAGAAGTCAAGAAACAGGTTACTCCTGTTTGAGTGAGGAAAAGTTGGTTTGCCTGTCTGTGGTCTTTTTATAATCTTTTTCTACAGAAGAGAAAGTGGGTAATTTTGTTTGAGAGTGGAAATATTCTCTAGTGGGAATCTACTAGGAGTAATTTATTTTCTATAAACTAAGTAAAGTTTGGAAGGTGACAAAAAGAAAGACAAAAATCTTGGAATTGTTTTAGACAACCAAGGTTTTCTTGCTCAGA**ATG…**

***SlZISO* (Solyc12g098710):**

CATATCCAAGCAATGGAAGAGAATGGTCACAAGAACAACCTCCCAAGTTATGCTTCACAAAAGCCCCTGTGAAAACAACCTTCCTTTTCAATGGTGTACATCCTAGTGTATAAATTTGATGTTAGGTGTGTTATTATTCTCTTGCTGTGAATCCAGGTAAAAGTCATTGTAATTTGGTCAGTCCTCCTCTGCTGGAAAAGCCTTGTGCTTTCAATGCTTGGTAGAATATAGAATCAAGTGAATAGTGTATCTTTTCGATCAATGTTTTGTGCTTGTAAGTTGAAAAAAAATGTACAATGAGGTTTTATATTGTATCCATTTATGGGATTGTACTTACTATTCACAACTTAGTTGATTTGACTCGACTTTCAAATCACTTGTATCGTATTCACGCGGTTTAGTGATTAATGAAATGAAAATAGGTTGATAACGATAAATGTTAGTATTCACGCGGTTTAGTGATTAATGAAATGAAAATAGGTTGATAACGATAAATGTTAGTGACGAATAATTCTAAAATTGCTGGCATACAGTATCACCCAAACAAATGGATATTTACGACAAATCAATTTTACTTATTGAACTTAGGTGTTTTATTATAATGAGAATAGGTACATTGAATTTATACATACATATCATATATGTATTTATTTTTATTAGACGTAAACACTTGATTGAACTTAGGTGTTTTATTATAATGAGAATAGGTACATTGAATTTATACATACATATCATATATGTATTTATTTTTATTAGACGTAAACACTTGATTGAACTTAGGTGTTTTATTATAATGAGAATAGGTACATTGAATTTATACATACATATCATATATGTATTTATTTTTATTAGACGTAAACACTTGATACAAGTAAATTTTTATCATTACTTTAAAAGTGTATATATATATATATGATTAGGTAAACATTATTTTGCCGACCCAAACCAAGAAAAAACAATAAAATTAGCTACAAGTAGTGTATCATTTGCCCACTTTCCTCCACTAGTTATCCAAAATGCCCACCAAAGAGCACAACTCCTTTAAGATACCTTTTTCCCCTTGCCTAATAAACTTGTTTTGGTCACAGTTTGTAAAAAAAAAAAATTAAATGTTTTTGAATAATAACATAAAAAATTATTTTTTTGAAAAGATGAAAAAAATAATTTCCTTTTAAAAATACTTGCAACAAATAAGATTTCTCAAAATAATTTTTTTTATGTCGTGTAATTTGATTTTCCACCTCGGAATCCTCTTTTCTAGTTACTCTACCCCCAATTTTTATTTATTTGTTGAAAAAAACTAAGAACTTGGCCAAGACAGGTTTATATATTTACCAAAACTACACACTTAGAGCTCACATAATCTTGTAAAA**ATG…**
